# DUSP5 and DUSP6, two ERK specific phosphatases, are markers of a higher MAPK signaling activation in *BRAF* mutated thyroid cancers

**DOI:** 10.1371/journal.pone.0184861

**Published:** 2017-09-14

**Authors:** Camille Buffet, Karine Hecale-Perlemoine, Léopoldine Bricaire, Florent Dumont, Camille Baudry, Frédérique Tissier, Jérôme Bertherat, Beatrix Cochand-Priollet, Marie-Laure Raffin-Sanson, Françoise Cormier, Lionel Groussin

**Affiliations:** 1 INSERM, U1016, Institut Cochin, Paris, France; 2 CNRS, UMR8104, Paris, France; 3 Université Paris Descartes, Sorbonne Paris Cité, France; 4 Department of Pathology, Pitié-Salpêtrière Hospital, Paris, France; 5 Department of Endocrinology, Cochin Hospital, Paris, France; 6 Department of Pathology, Cochin Hospital, Paris, France; 7 Department of Endocrinology, Ambroise-Paré Hospital, Boulogne-Billancourt, France; Medical College of Wisconsin, UNITED STATES

## Abstract

**Background:**

Molecular alterations of the MAPK pathway are frequently observed in papillary thyroid carcinomas (PTCs). It leads to a constitutive activation of the signalling pathway through an increase in MEK and ERK phosphorylation. ERK is negatively feedback-regulated by Dual Specificity Phosphatases (DUSPs), especially two ERK-specific DUSPs, DUSP5 (nuclear) and DUSP6 (cytosolic). These negative MAPK regulators may play a role in thyroid carcinogenesis.

**Methods:**

MAPK pathway activation was analyzed in 11 human thyroid cancer cell lines. Both phosphatases were studied in three PCCL3 rat thyroid cell lines that express doxycycline inducible PTC oncogenes (RET/PTC3, H-RAS^V12^ or BRAF^V600E^). Expression levels of DUSP5 and DUSP6 were quantified in 39 human PTCs. The functional role of DUSP5 and DUSP6 was investigated through their silencing in two human BRAF^V600E^ carcinoma cell lines.

**Results:**

BRAF^V600E^ human thyroid cancer cell lines expressed higher phospho-MEK levels but not higher phospho-ERK levels. DUSP5 and DUSP6 are specifically induced by the MEK-ERK pathway in the three PTC oncogenes inducible thyroid cell lines. This negative feedback loop explains the tight regulation of p-ERK levels. DUSP5 and DUSP6 mRNA are overexpressed in human PTCs, especially in BRAF^V600E^ mutated PTCs. DUSP5 and/or DUSP6 siRNA inactivation did not affect proliferation in two BRAF^V600E^ mutated cell lines, which may be explained by a compensatory increase in other phosphatases. In the light of this, we observed a marked DUSP6 upregulation upon DUSP5 inactivation. Despite this, DUSP5 and DUSP6 positively control cell migration and invasion.

**Conclusions:**

Our results are in favor of a stronger activation of the MAPK pathway in BRAF^V600E^ PTCs. DUSP5 and DUSP6 have pro-tumorigenic properties in two BRAF^V600E^ PTC cell line models.

## Introduction

In papillary thyroid carcinoma (PTC), the most common thyroid malignancy, activating-mutations of genes encoding effectors of the mitogen-activated protein kinase (MAPK) pathway are central for malignant transformation. This pathway transduces mitogenic signals via activation of receptor tyrosine kinases, leading to the successive recruitment and activation of RAS and members of the RAF family of serine/threonine kinases. A cascade of phosphorylation is initiated, culminating in the activation by phosphorylation of MEK and consequently of the extracellular signal-regulated kinases (ERK).

Rearrangements of the gene encoding the receptor tyrosine kinase *RET* and activating-point mutations of *RAS* or *BRAF* are found in 70% of all cases of PTC and are mutually exclusive. Mutation of *BRAF*, especially a valine to glutamic acid substitution at codon 600 (V600E) termed the BRAF^V600E^ mutation, is the most frequent genetic alteration, identified in 60% of cases (range: 27%-87%) [1, 2]. PTCs with different mutations are recognized to have distinct histopathologic appearance and biologic properties. Most studies in PTCs suggest that *BRAF* activating mutation is closely associated with extrathyroidal extension, lymph node metastasis, advanced tumor stages, disease recurrence, and even patient mortality [2]. The molecular mechanisms underlying the association of *BRAF* mutation with worse prognosis compared to PTC with no or other genetic alterations has so far only been partially elucidated. There is evidence that the transcriptional output of the MAPK pathway is greatest in BRAF^V600E^ mutant tumors, because this oncoprotein signals as a monomer and is therefore unresponsive to feedback inhibition on RAF dimers [3].

Levels of ERK phosphorylation (p-ERK) are dictated by the coordinated activities of protein kinases and phosphatases. Identical p-ERK immunostaining levels described in human PTC regardless of the genetic alteration [4, 5] could be explained by negative feedback control implementing at the p-ERK level. Thus, we sought to study two dual-specificity (Thr/Tyr) MAPK phosphatases (DUSPs), DUSP5 and DUSP6, which belong to this large family known to act as central feedback regulators attenuating MAPK signaling. Both phosphatases are induced by ERK signaling and specifically inactivate ERK by dephosphorylation [6–8]. DUSP5 localizes in the nucleus [9] while DUSP6 is in the cytoplasm [10]. Complex post-translational regulation of both phosphatases allows p-ERK steady state levels to be tightly regulated. Accumulation of DUSP5 and DUSP6 protein is regulated by rapid proteasomal degradation [6, 11]. Accordingly, increasing levels of p-ERK will induce DUSP5 and DUSP6 expression, which in turn will be rapidly degraded.

*DUSP6* is a candidate tumor suppressor gene, especially in pancreatic cancers, which have a high prevalence of *KRAS* mutations [8]. In PTCs, data from transcriptional expression profile studies suggest that *DUSP5* and *DUSP6* are overexpressed [1, 12]. DUSP6 may have a pro-tumorigenic role in thyroid carcinogenesis, as recent data demonstrated that DUSP6 silencing reduced the neoplastic properties of human thyroid carcinoma cell lines with different genetic background (RET/PTC1 and BRAF^V600E^) [13]. Although DUSP5 is the lead candidate to serve as the critical site for mitogenic signal termination and sequestration of ERK away from MEK, its cytoplasmic activator, published data on its role in carcinogenesis are scarce [8]. The functional and clinical significance of DUSP5 mediated regulation of ERK signaling in PTCs has not been investigated.

We hypothesized that DUSP5 and DUSP6 may be biomarkers of the MAPK output in PTCs, in the context of a negative feedback loop. Alternatively, they could act as tumor suppressor genes. For that purpose, we studied the MAPK pathway activation, the expression and regulation of DUSP5 and DUSP6, and the consequences of their inactivation in thyroid cancer cell lines. We found out that DUSP5 and DUSP6 are overexpressed in human thyroid carcinomas and are surrogate markers of MAPK pathway activation. Silencing of DUSP5 or DUSP6, or both phosphatases in thyroid cell lines does not affect proliferation, possibly explained by compensation between phosphatases of the DUSP family. Finally, DUSP5 and DUSP6 silencing reduced the cell migration and invasion capacities of two BRAF^V600E^ thyroid cancer cell lines, thus suggesting a pro-tumorigenic role of these phosphatases in PTC.

## Materials and methods

### Cell lines

PCCL3 RET/PTC3, HRAS^V12^ and BRAF^V600E^ are three cells lines derived from the well differentiated, non-transformed rat thyroid cell line PCCL3 that conditionally express one of the three known thyroid oncogenes (RET/PTC3, HRAS^V12^ or BRAF^V600E^), in a doxycycline-dependent manner [14–16]. These cell lines were propagated in H4 complete medium, as previously described [14].

We used a panel of 11 human thyroid cancer cell lines. The 8505c (DSMZ number: ACC 219) and B-CPAP (DSMZ number: ACC 273) cell lines were purchased from the German Collection of Microorganisms and Cell Culture. This cell bank genetically fingerprinted these last two cell lines by multiplex PCR of minisatellite markers that revealed a unique DNA profile. All other cell lines were provided by J. A. Fagin (Memorial Sloan-Kettering Cancer Center, New York, USA) in 2010 and used with permission from: N.-E. Heldin (University Hospital, Uppsala, Sweden): HTh74 [17], C643 [18], SW1736 [19]; N. Onoda (Osaka University of Medicine, Osaka, Japan): TTA-1 [20] and ACT-1 [21]; S.M. Jhiang (Department of Physiology and Cell Biology, the Ohio State, USA): TPC-1 [22]; M. Santoro (Department of Biology and Cellular and Molecular Pathology, Institute of Endocrinology and Experimental Oncology National Research Council, Naples, Italy): Cal62 [23, 24]; J. Kurebayashi (Departments of Breast and Thyroid Surgery, Kawasaki Medical School, Kurashiki, Okayama, Japan): KTC1 [25]; and G. Andrew (Rudbeck Laboratory, Uppsala University, Uppsala, Sweden): Hth104 [26]. C643 and Cal-62 have been DNA profiled in 2010 [24] by short tandem repeat (STR) analysis and shown to be unique and identical to those reported by Schweppe et al. [27]. Other cell lines were genetically fingerprinted by either single nucleotide polymorphism comparative genomic hybridization or polymorphic short tandem repeat and verified to be unique in 2008 [27]. All cell lines were passaged in our laboratory for fewer than 3 months after resuscitation and were cultured according to the manufacturer’s recommendations.

### Human thyroid samples

Formalin fixed paraffin-embedded (FFPE) PTC and adjacent normal thyroid specimens were obtained from the Departments of Pathology at the Cochin and Ambroise-Paré University Hospitals in Paris, France. Informed signed consent for genetic diagnosis, tumor analysis, and for access to the data collected was obtained from all the patients. Total RNA was extracted from a 10-μm section of the FFPE tumor tissue using TRIzol Reagent (Life technologies, Carlsbad, CA, USA) according to the manufacturer’s protocol and 1μg was reverse transcribed to generate cDNA using High-Capacity^®^ cDNA Reverse Transcription Kit (Applied Biosystems, Foster City, CA, USA).

Exon 14 and 15 of the *BRAF* gene and exon 1 and 2 of the *HRAS*, *NRAS*, and *KRAS* genes were amplified. Direct sequencing of the purified fragments was then performed using the Genetic Sequencer ABI3100 Applied Biosystems apparatus. Sequence comparisons were carried out using the Chromas Program.

For RET/PTC rearrangements, unbalanced expression of exons 12 and 13 relative to exons 10 and 11 of RET were searched, by quantitative real-time PCR (qPCR) using LightCycler^®^ 480 Real-Time PCR System (Roche Applied Sciences, Indianapolis, USA) as already described [28]. Then, the presence of RET/PTC1, RET/PTC2 or RET/PTC3 rearrangement was assessed using nested PCR with appropriate primers.

Relative expression of *DUSP5* and *DUSP6* was calculated for all tumors using the ΔΔCt method and GAPDH as an internal control.

### Reagents

The MEK inhibitor, UO126 (Cell Signaling Technology, Danvers, USA) and selumetinib AZD6244 (Selleck Chemicals) and the PI3K inhibitor, LY294002 (Euromedex, Mundolsheim, France) were used with the indicated concentration.

### Western blotting

Cells were lysed in a buffer containing 20 mmol/L Tris-HCl (pH 7.5), 1mmol/L EDTA, NaCl 150 mM, NP40 1%) phosphatase (PhosSTOP^™^, Roche) and protease inhibitor cocktails (cOmplete^™^, Mini Protease Inhibitor Cocktail, Roche). Proteins were quantified using the Bradford protein assay and separated by SDS-polyacrilamide gel (10%), then transferred onto nitrocellulose membrane.

The following primary antibodies were used: mouse monoclonal anti-BRAF^V600E^ (catalog #: 790–4855, Roche, Ventana Medical Systems, Inc., Tucson, Arizona, USA), mouse monoclonal anti-p-ERK (catalog #: sc-7383; Santa Cruz Biotechnology, Inc., Santa Cruz, USA), rabbit polyclonal anti-p-MEK 1/2 (catalog #: 9121, Cell Signaling Technology), rabbit polyclonal anti-total-ERK (catalog #: 06–182, Cell Signaling Technology), rabbit polyclonal anti-total-MEK1 (catalog #: sc-219; Santa Cruz Biotechnology, Inc.), rabbit polyclonal anti-total-MEK2 (catalog #: 9125, Cell Signaling Technology), mouse monoclonal anti-DUSP6 (catalog #: H00001848-M01, Abnova, Taipei, Taiwan) and rabbit polyclonal anti-GAPDH (catalog #: sc-25778, Santa Cruz Biotechnology, Inc.). Primary antibody against DUSP5 was provided by Stephen Keyse [9]: it is a polyclonal antibody raised in sheep against the full-length recombinant protein expressed in E. coli. This antibody was validated with positive and negative controls, derived from wild type and DUSP5-/- mice. The antigen-antibody complexes were visualized using secondary horseradish peroxidase-conjugated anti-mouse (Santa Cruz Biotechnology, Inc.) and anti-rabbit (Cell Signaling Technology) antibodies and enhanced chemiluminescence system. Staining intensity was quantified using the Image J software.

### RNA extraction, reverse transcription and qPCR

Total RNA (500 ng to 1μg) extracted from PCCL3 cells and human cell lines by RNeasy Mini Kit (QIAGEN Inc., Austin, Texas) was reverse transcribed to generate cDNA using High-Capacity^®^ cDNA Reverse Transcription Kit (Applied Biosystems). LightCycler^®^ 480 Real-Time PCR System (Roche) was used for qPCR. The following primer pairs were used: DUSP5 (5’-GCC CGC GGG TCT ACT TCC TCA AA-3’ and 5’-ATT TCA ACT GGG CCA CCC TGG-3’), DUSP6 (5’- TCC CTG AGG CCA TTT CTT TCA TAG ATG-3’ and 5’-GCA GCT GAC CCA TGA AGT TGA AGT-3’), GAPDH (5’- GCC ACA TCG CTC AGA CAC CA -3’ and 5’- TTC CCG TTC TCA GCC TTG AC -3’) and cyclophilin (5’- ATGGCACTGGTGGCAAGTCC-3’ and 5’-TTGCCATTCCTGGACCCAAA-3’). Relative expression of *DUSP5* and *DUSP6* was calculated using the ΔΔCt method and the cyclophilin or GAPDH as an internal control.

### Small interfering RNA transfection

For small interfering RNA (siRNA) experiments, siRNA for human *DUSP5* (si*DUSP5* (1): 5’-GGCCTTCGATTACATCAAG- 3’, si*DUSP5* (2): 5’-GAGAAGATTGAGAGTGAGA- 3’, si*DUSP5* (3); 5’- GGAAGUGCCUACCAUGCAU- 3’) for human DUSP6 (si*DUSP6* (1): 5’-GAACTGTGGTGTCTTGGTA-3’ si*DUSP6* (2): 5’- GGAATTCGGCATCAAGTAC- 3’), and a control siRNA (5’- GGCATAGATGTAGCTGTAA- 3’) were purchased from Eurofins MWG Operon. siRNAs were transfected at the concentration of 100 nmol/L with Lipofectamine Plus (Invitrogen, Carlsbad, USA). After transfection, the suppression of targeted proteins was determined by reverse transcription qPCR and Western blotting. Cell viability was determined as described below.

### Proliferation assay

Thiazolyl Blue Tetrazolium Bromide (MTT) (Sigma-Aldrich) was dissolved in PBS at 5 mg/ml and filtered. Cells were seeded in 96-well plates and incubated at 37°C. Stock MTT (5mg/ml) solution (10 μ1 per 100μ1 medium) was added to all wells (10 wells per condition in each experiment) of an assay. After 4 hours of additional incubation, 100 μl of 0.04 N HCI in isopropanol was added to each well. Plates were then read within 1 hour on an automatic enzyme linked immunosorbent assay plate reader (Tecan Austria GmbH, Männedorf, Switzerland). The optical density was calculated as the difference between the absorbance at a reference wavelength (690 nm) and that at a test wavelength (570nm) corrected to the blanks. Within each experiment, the means and the standard errors of the means were calculated.

### Cell migration and invasion assay

Forty-eight hours after transfection with DUSP5 or DUSP6 siRNA, cells were suspended in 1% foetal calf serum (FCS) DMEM medium into the upper chamber of 24-well transwell chambers (Costar, Corning, Inc., Corning, NY, USA). Previously DMEM medium containing 10% FCS were added to the lower chambers. For the invasion assay, the transwell membranes were coated with Growth Factor Reduced Matrigel (12.5 μg in 60 ml/well) (BD Biosciences) and dried for 1 h. Cells were transferred onto the artificial basement membrane.

After overnight incubation at 37°C, cells that invaded the Matrigel layer and/or migrated to the lower chamber were fixed in methanol, stained with Giemsa and counted under an inverted microscope. Assays were performed in triplicate, cells were either counted in adjacent fields (n = 9) and data were reported as average cell number per field or with an Image J software macro and data were reported as arbitrary density unit.

### Anchorage-independent growth assay

The anchorage-independent growth was analyzed through a soft agar colony formation assay. Twenty four hours after transfection with siRNA, cells (25.10^3^ 8505C cells or 30.10^3^ BCPAP cells) were seeded on 6-well plates in 0.3% agar in culture medium over a base layer of 0.7% agar, Colonies were microscopically counted after 3 weeks of culture.

### Statistical analysis

Wilcoxon and paired t tests were used to compare *DUSP5* and *DUSP6* mRNA levels in human PTC and normal adjacent thyroid tissues. Wilcoxon test was used to compare p-MEK/t-MEK, p-ERK/t-ERK levels in BRAF^V600E^ human PTC cell lines *vs* the other cell lines. Bioinformatic analysis of The Cancer Genome Atlas (TCGA) [29] Reverse Phase Protein Lysate Microarray (RPPA) were performed by Florent Dumont (Cochin Institute bioinformatics platform) and Leanne De Koning (RPPA platform, Institut Curie, France). Bioinformatic analysis of TCGA transcriptomic data was performed by Florent Dumont with the same previously published methodology [29]. Unpaired t-tests were used to compared TCGA RPPA data, migration/invasion and anchorage-independent growth assay data. Anova tests were used to compare TCGA transcriptomic data.

## Results

### Higher MAPK pathway activation in BRAF^V600E^ mutated thyroid cell lines

**Fig 1:**
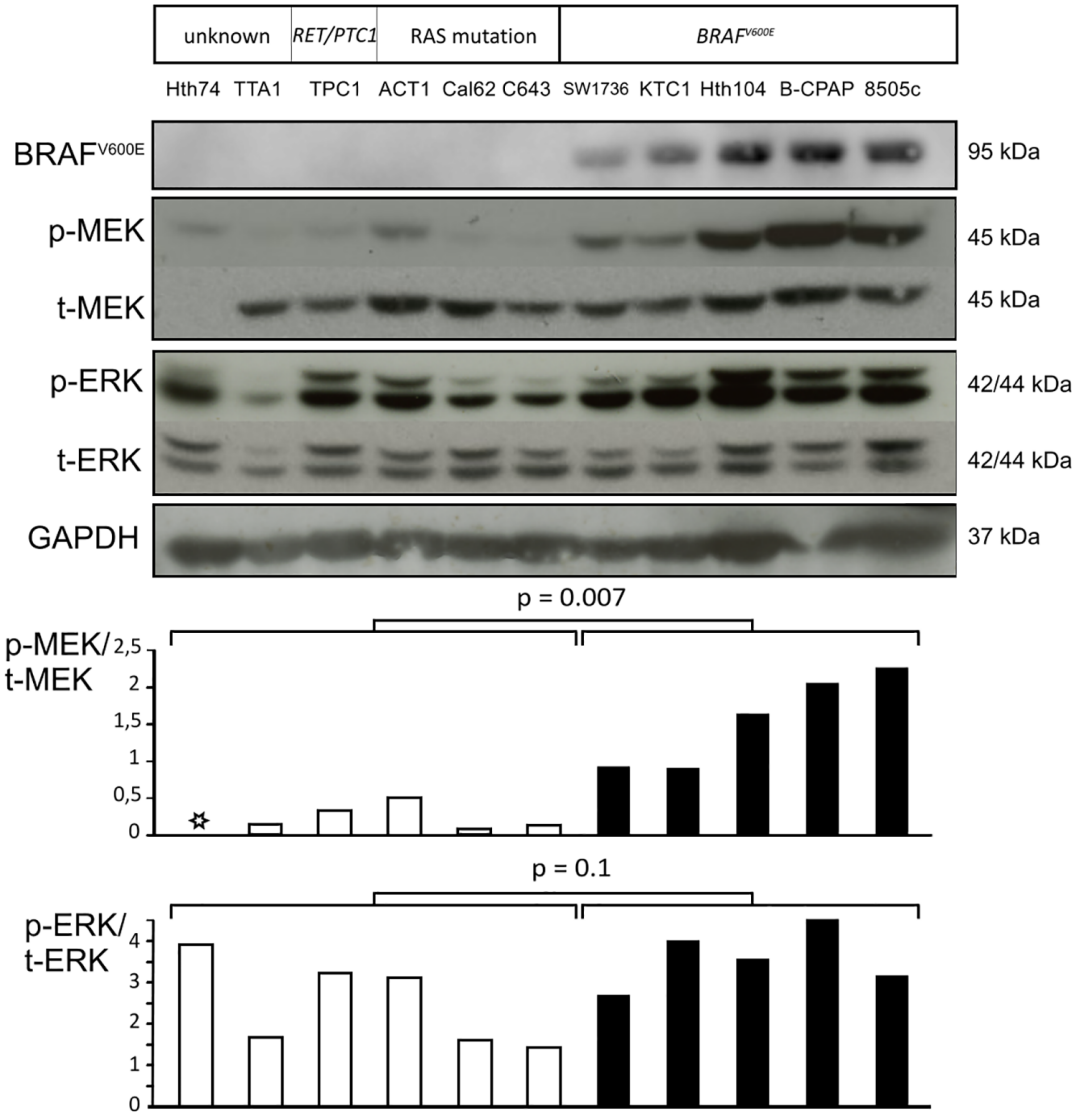
High phosphorylated MEK levels in human thyroid carcinoma cell lines harboring the BRAF^V600E^ mutation. Eleven human thyroid cancer-derived cell lines were grown in 10% FBS medium and studied for MEK and ERK activation: two cell lines without known MAPK pathway genetic alteration, one with a *RET/PTC* rearrangement, two with a *RAS* activating point mutation, and five with the BRAF^V600E^ mutation. Protein expression levels were assayed by immunoblot for the BRAF^V600E^ mutation, phosphorylated MEK (p-MEK), total MEK1 (t-MEK1), phosphorylated ERK (p-ERK), total ERK (t-ERK) and GAPDH. Graphs represent subsequent quantification of p-MEK/t-MEK1 and p-ERK/t-ERK ratios. The black star on the upper graph indicates that the quantification of p-MEK/t-MEK1 was not done in the Hth74 cell line due to the loss of t-MEK1 expression. However, MEK2 expression was secondary assessed and is preserved explaining the p-MEK band (data not shown).

Activation of the MAPK signaling pathway leads to a kinase cascade culminating downstream in the phosphorylation of ERK by MEK. We analyzed 11 human thyroid carcinoma cell lines representing the known MAPK pathway molecular alterations in PTCs. Among them, 5 were BRAF^V600E^ mutated cell lines, as confirmed by Western Blot with a sensitive and specific anti-BRAF^V600E^ antibody (Fig 1). All cell lines displayed detectable p-ERK levels, which although variable did not differ significantly (p = 0.1) between BRAF wild-type and BRAF^V600E^ mutated cell lines (Fig 1). In contrast, p-MEK/total-MEK ratios (Fig 1) were markedly elevated in BRAF^V600E^ mutated cell lines in comparison with others (p = 0.007), suggesting a higher MAPK pathway activation by BRAF^V600E^. The same results were obtained with serum-starved (i.e. 0.5% FBS) cells (S1 Fig).

We did not find a significant correlation between MAPK activation i.e. phospho-MEK levels and DUSPs expression at the mRNA and protein levels in our 11 human thyroid cancer cell lines (S2 Fig). However it is difficult to draw any conclusion given the insufficient number of RAS, RET/PTC and “unknown” cell lines in comparison with the five BRAF^V600E^ mutated cell lines.

Since p-ERK levels tend to be the same regardless of the genetic alteration, we assumed that the steady state of p-ERK levels would be maintained by high levels of feedback inhibitors that dephosphorylate ERK and allow tight regulation of p-ERK levels. Accordingly, we studied the regulation of DUSP5 and DUSP6, known to specifically dephosphorylate and inactivate ERK.

### Induction of *DUSP5* and *DUSP6* expression in doxycycline-inducible PCCL3 cell lines and differential MAPK pathway activation according to the genetic alterations

**Fig 2:**
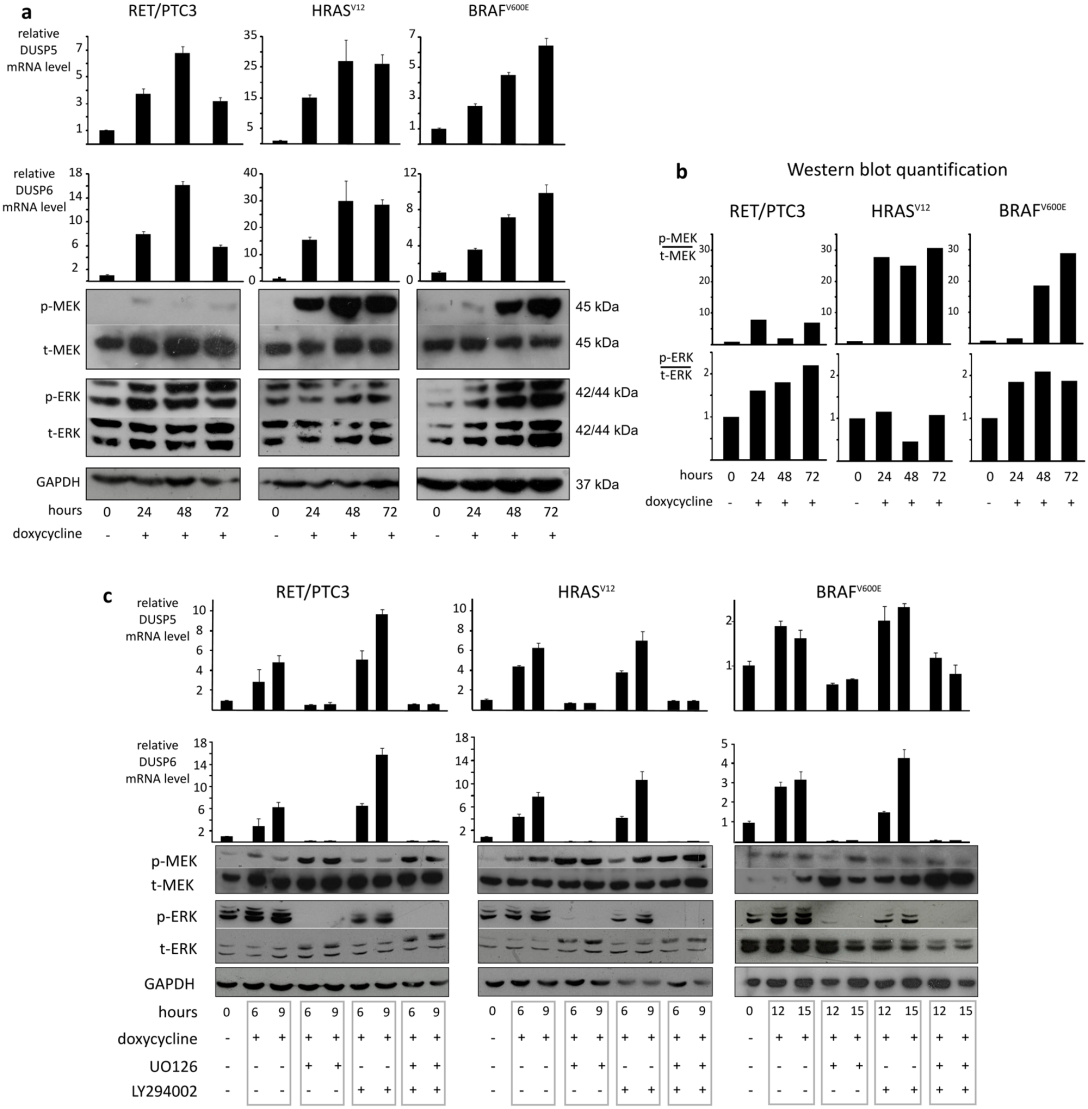
Induction of DUSP5 and DUSP6 expression by the MAPK pathway in PCCL3 cell lines. (a) Real-time Reverse-Transcription qPCR showing the expression levels of *DUSP5* and *DUSP6* mRNA in three PCCL3 cell lines harboring either RET/PTC3 or HRAS^V12^ or BRAF^V600E^. Oncogene expression was induced by doxycycline treatment (1μg/ml) for 72 hours. Results are normalized to *cyclophilin* mRNA levels. Basal *DUSP5* and *DUSP6* mRNA levels were arbitrary set at 1. Lysates of the three PCCL3 cell lines were subjected to immunoblotting with the indicated antibodies. (b) Graphs represent subsequent quantification of p-MEK and p-ERK levels of the aforementioned western blot. (c) The three PCCL3 cell lines were treated for the indicated times with doxycycline alone or in combination with the MEK inhibitor UO126 (20μM) and/or the PI3K inhibitor LY294002 (10μM). *DUSP5* and *DUSP6* mRNA were then analyzed using real time Reverse-Transcription qPCR and normalized to *cyclophilin* mRNA levels. *DUSP5* and *DUSP6* mRNA levels before doxycycline treatment were arbitrary set at 1. Lysates of the three PCCL3 cell lines were subjected to immunoblotting with the indicated antibodies.

MAPK pathway-dependent regulation of these phosphatases was analyzed in three rat PCCL3 thyroid cell lines that conditionally express one of the three following known thyroid oncogenes (RET/PTC3, HRAS^V12^ or BRAF^V600E^**)**, in a doxycycline-dependent manner. We first demonstrated that a strong induction of oncogenic RET/PTC3, HRAS^V12^ or BRAF^V600E^ was correlated with a marked increase in *DUSP5* and *DUSP6* mRNA levels compared to baseline during a 72 hours kinetic of doxycycline treatment (Fig 2a).

In parallel with doxycycline-inducible expression of oncogenic RET/PTC3, HRAS^V12^ or BRAF^V600E^, p-ERK levels increased compared to baseline, though slightly, remain relatively constant and did not differ significantly between the three cell lines during the 72 hours kinetic (Fig 2a and 2b). However, levels of p-MEK were strongly elevated in PCCL3 cells with BRAF^V600E^ and HRAS^V12^ compared with those with RET/PTC3 rearrangement (Fig 2a and 2b). These results suggest differential MAPK pathway activation according to the type of oncogene. The levels of p-MEK seem to be better correlated with enhanced activation of the MAPK pathway than p-ERK levels. In parallel, we demonstrated that doxycycline itself had no effect on ERK phosphorylation in non-transformed PCCL3 cells (S3 Fig).

### Regulation of *DUSP5* and *DUSP6* mRNA by the MAPK pathway in PCCL3 cell lines

Using specific inhibitors, namely UO126 (MEK inhibitor) and LY294002 (PI3K inhibitor), we tested the involvement of the ERK1/2 and PI3K/Akt pathways respectively, in the up-regulation of *DUSP5* and *DUSP6* mRNA in the three PCCL3 cell lines. Once again, we observed after doxycycline-inducible expression of RET/PTC3, HRAS^V12^ or BRAF^V600E^, a marked and rapid increase of *DUSP5* and *DUSP6* mRNA in parallel to ERK activation (Fig 2c). *DUSP5* and *DUSP6* induction was blocked by MEK inhibition, resulting in a decrease in ERK phosphorylation (Fig 2c). On the other hand, PI3K inhibition could not prevent the *DUSPs* up-regulation. Furthermore, after UO126 treatment, MEK phosphorylation was increased in RET/PTC3 and HRAS^V12^ cell lines suggesting a relief of ERK feedback inhibition upstream of MEK (Fig 2c). This was not observed in the BRAF^V600E^ PCCL3 cell line. These data argue that BRAF^V600E^ mutated cells are insensitive to relief of ERK dependent upstream negative feedback. Similarly, in BRAF wild-type human PTC cell lines MEK phosphorylation was clearly increased after MEK inhibition (i.e. treatment with AZD6244) (S4 Fig). On the opposite steady-state levels of pMEK were elevated in BRAF-mutated cells in comparison with BRAF-wild type cells and MEK inhibition does not further induce p-MEK levels in BRAF^V600E^ cells (S4 Fig).

**Fig 3:**
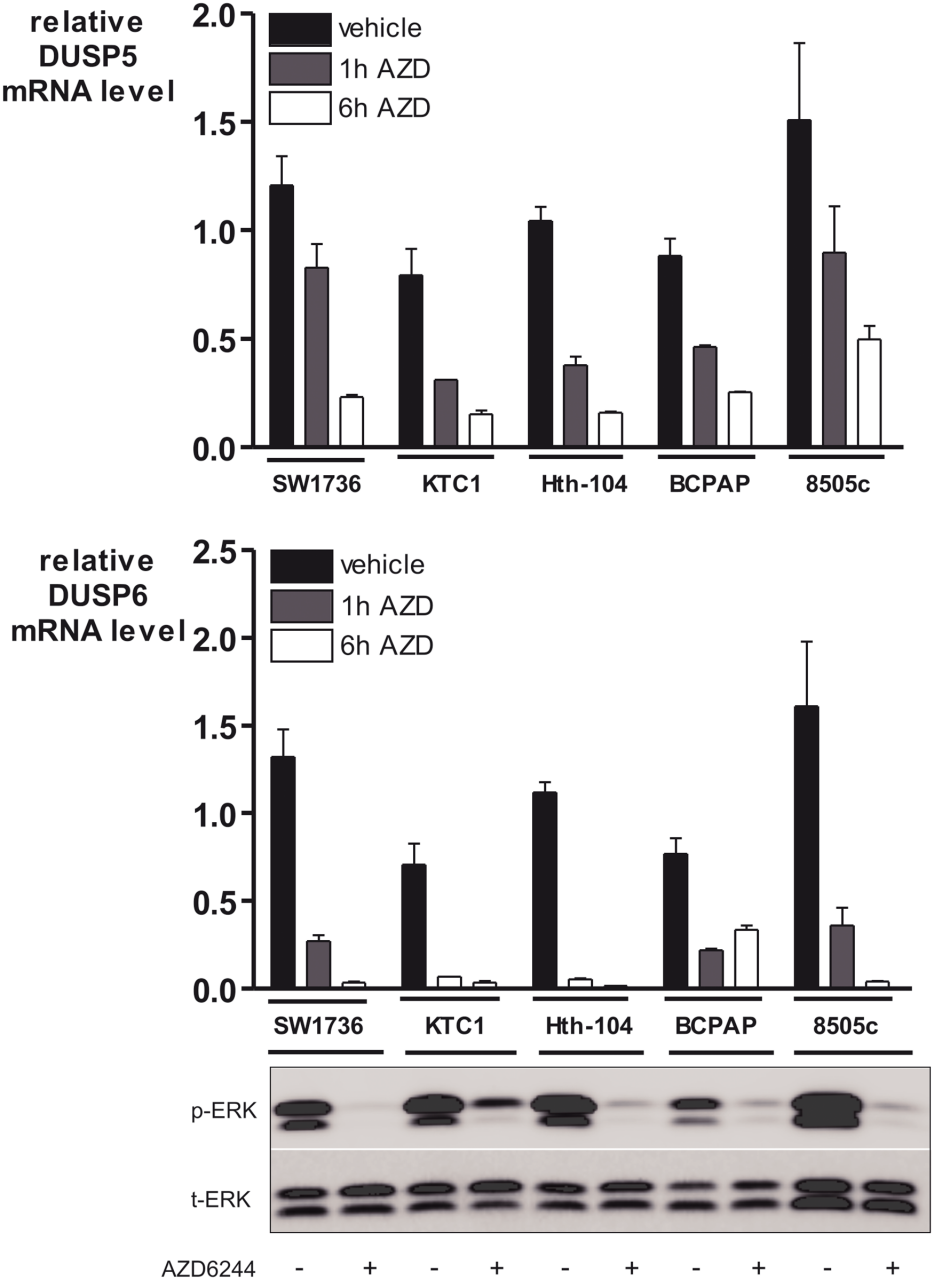
DUSP5 and DUSP6 are regulated by the MAPK pathway in BRAF-mutated human thyroid carcinoma cells. The five BRAF^V600E^-mutated human thyroid carcinoma cell lines were treated for 1h and 6h with the MEK inhibitor AZD6244 (selumetinib). *DUSP5* and *DUSP6* mRNA were then analyzed using real time Reverse-Transcription qPCR and normalized to *cyclophilin* mRNA levels. Lysates of the five BRAF^V600E^-mutated human thyroid carcinoma cell lines were subjected to immunoblotting with p-ERK and t-ERK antibodies following treatment of cells for 6h with AZD6244 to confirm the decrease in pERK levels.

Furthermore we confirmed in our five BRAF-mutated human thyroid carcinoma cell lines that *DUSP5* and *DUSP6* induction was blocked by MEK inhibition, using a highly potent and selective MEK inhibitor namely AZD6244 [30] (Fig 3).

### *DUSP5* and *DUSP6* are overexpressed in papillary thyroid carcinomas (PTCs), especially in *BRAF* mutated tumors

**Fig 4:**
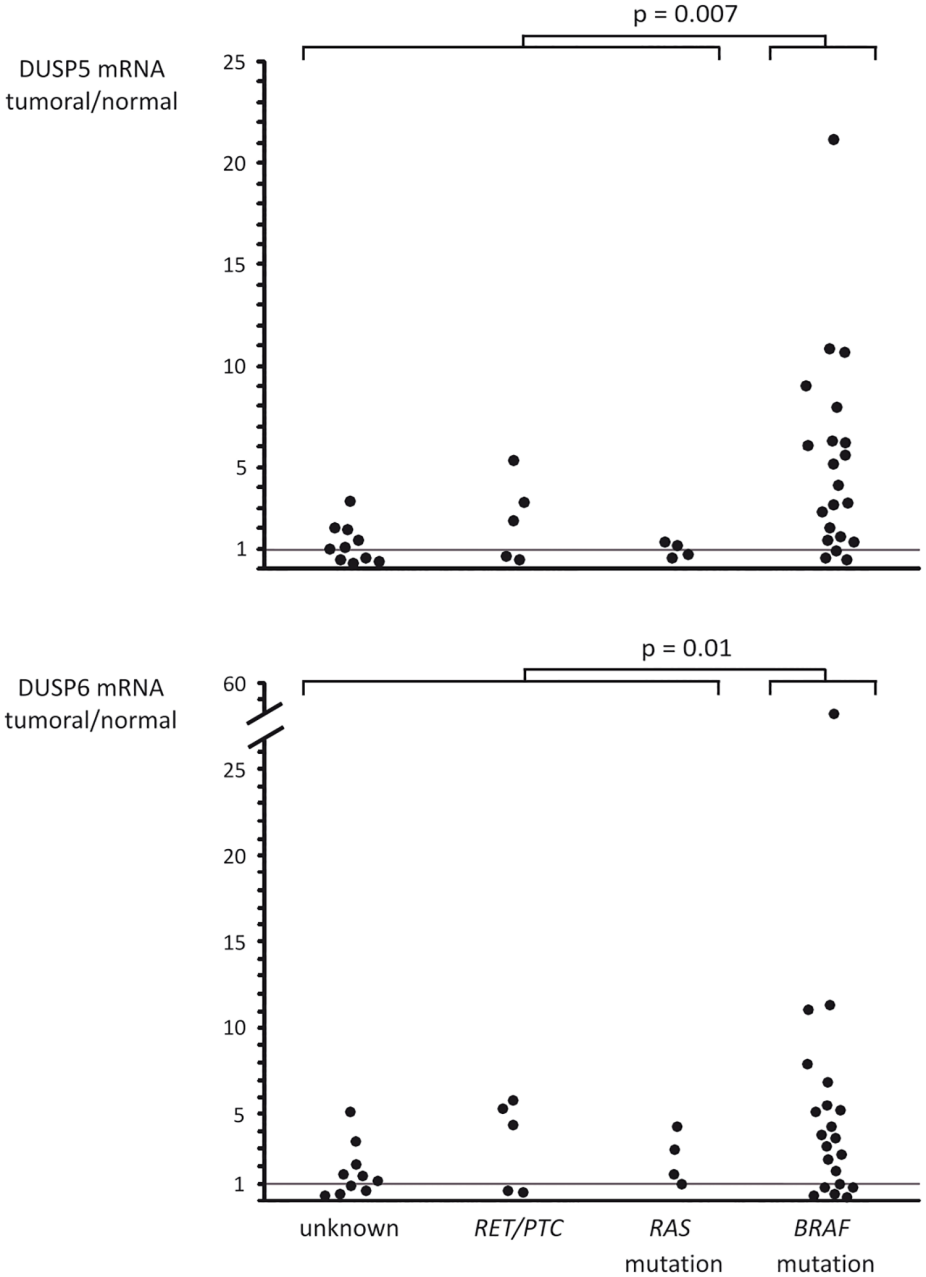
*DUSP5* and *DUSP6* are overexpressed in papillary thyroid carcinomas (PTCs), especially in BRAF^V600E^ mutated tumors. Real-time reverse transcription qPCR showing the expression levels of *DUSP5* and *DUSP6* in 39 human PTCs. Each dot represents the ratio between *DUSP5* or *DUSP6* mRNA levels in tumoral to normal adjacent thyroid tissue (mRNA expression normalized for *GAPDH* mRNA levels).

We showed in the PCCL3 cell lines that the MAPK pathway drives the expression of *DUSP5* and *DUSP6*. We hypothesized that *DUSP5* and *DUSP6* mRNA levels might be a marker for MAPK pathway activation, with differences according to the genetic alteration present in the carcinoma. To test this hypothesis, DUSPs expression were quantified by qPCR in a total of 39 human PTCs, characterized for known MAPK pathway genetic alterations (twenty *BRAF* mutated, four *RAS* mutated, five *RET/PTC* rearranged PTCs (two RET/PTC1, two RET/PTC3 and one with an undetermined RET/PTC rearrangement) and ten PTCs with no identified genetic alteration) and their normal adjacent thyroid tissue. *DUSPs* mRNA levels were found overexpressed in PTCs compared to normal adjacent thyroid tissue, with an average ratio of 1.43 for *DUSP5* (p = 0.001) and 2.26 for *DUSP6* (p = 0.001). *DUSP5* and *DUSP6* mRNA are statistically overexpressed (p = 0.007 and 0.01 respectively) in *BRAF*-mutated tumors in comparison with all tumors harboring either *RET/PTC* rearrangement or *RAS* mutation or none of these genetic alterations (Fig 4). To go further in the comparison between DUSP5 and DUSP6 mRNA levels according to the BRAF status, we analyzed TCGA transcriptomic data [29]. DUSP5 mRNA levels were 7.9 fold higher in BRAF-mutated papillary thyroid carcinoma (PTC) than in normal adjacent tissue (*P* = 6.3.10^−58^) and 1.5 fold higher in RAS-mutated PTC than in normal adjacent tissue (*P* = 8.10^−3^). DUSP6 mRNA levels were 6.7 fold higher in BRAF-mutated PTC than in normal adjacent tissue (*P* = 3.3.10^−87^) and 2.2 fold higher in RAS-mutated PTC than in normal adjacent tissue (*P* = 1.6.10^−10^).

**Fig 5:**
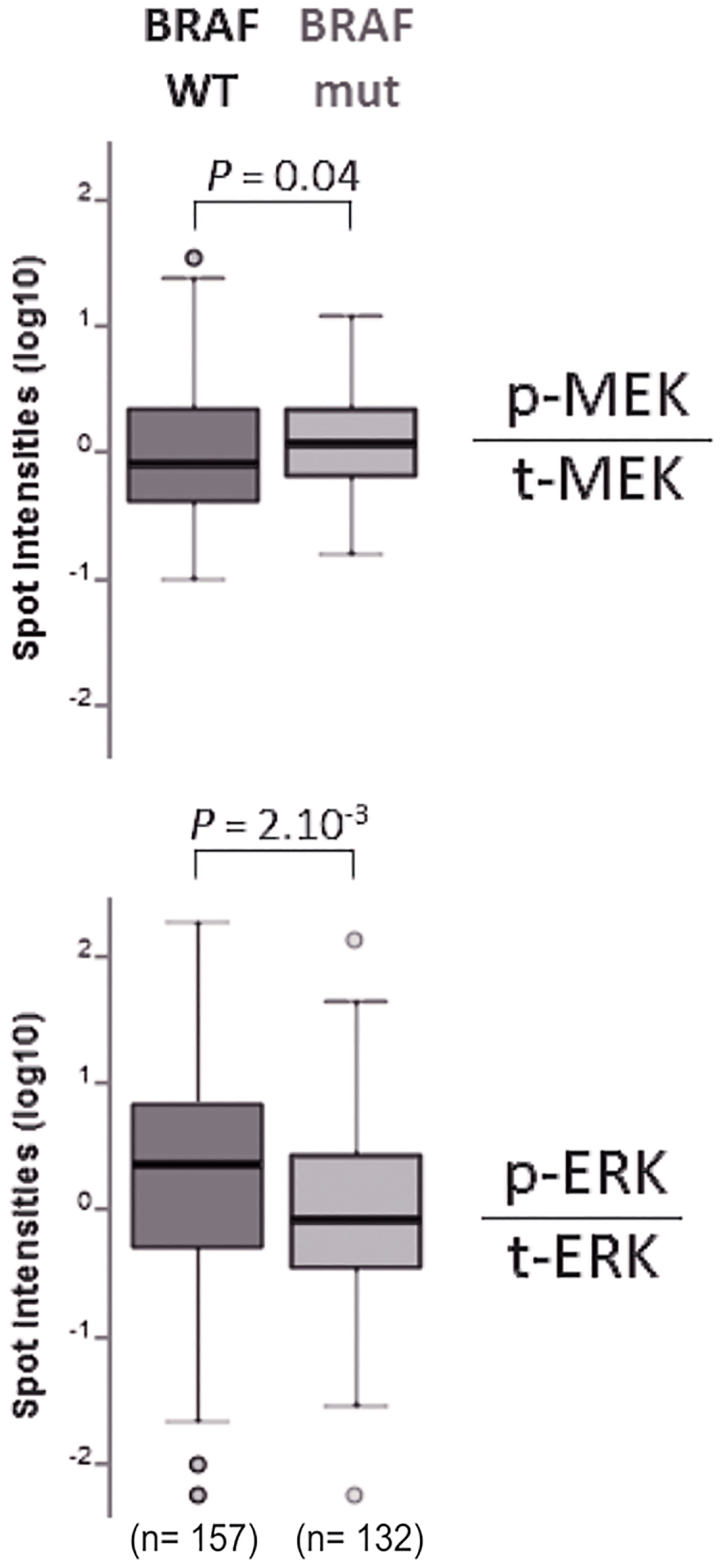
p-MEK/t-MEK and p-ERK/t-ERK ratios in 289 papillary thyroid carcinomas from The Thyroid Cancer Genome Atlas. Ratios were analyzed according to the presence or not of a BRAF mutation. Paired t-tests were used to compare the ratios between the two groups.

To determine if DUSPs overexpression in BRAF mutated PTCs correlates with a MAPK pathway overactivation, we performed a bioinformatic analysis of the recently published Reverse Phase Protein Lysate Microarray (RPPA) data from TCGA[29]. We compared the p-MEK/t-MEK and p-ERK/t-ERK ratios between wild-type and BRAF-mutated PTCs. A total of 289 samples were analyzed with RPPA in TCGA publication [29]. In BRAF-mutated PTCs p-MEK/t-MEK ratios were slightly higher (1.1 fold) but significantly different than in PTCs with other genetic alteration; p-ERK/t-ERK ratios were statistically significantly lower than in PTCs with other genetic alteration (Fig 5).

### Silencing of DUSP5 or DUSP6 or both phosphatases by siRNA does not affect viability of human BRAF^V600E^ papillary or anaplastic thyroid carcinoma cell lines

**Fig 6:**
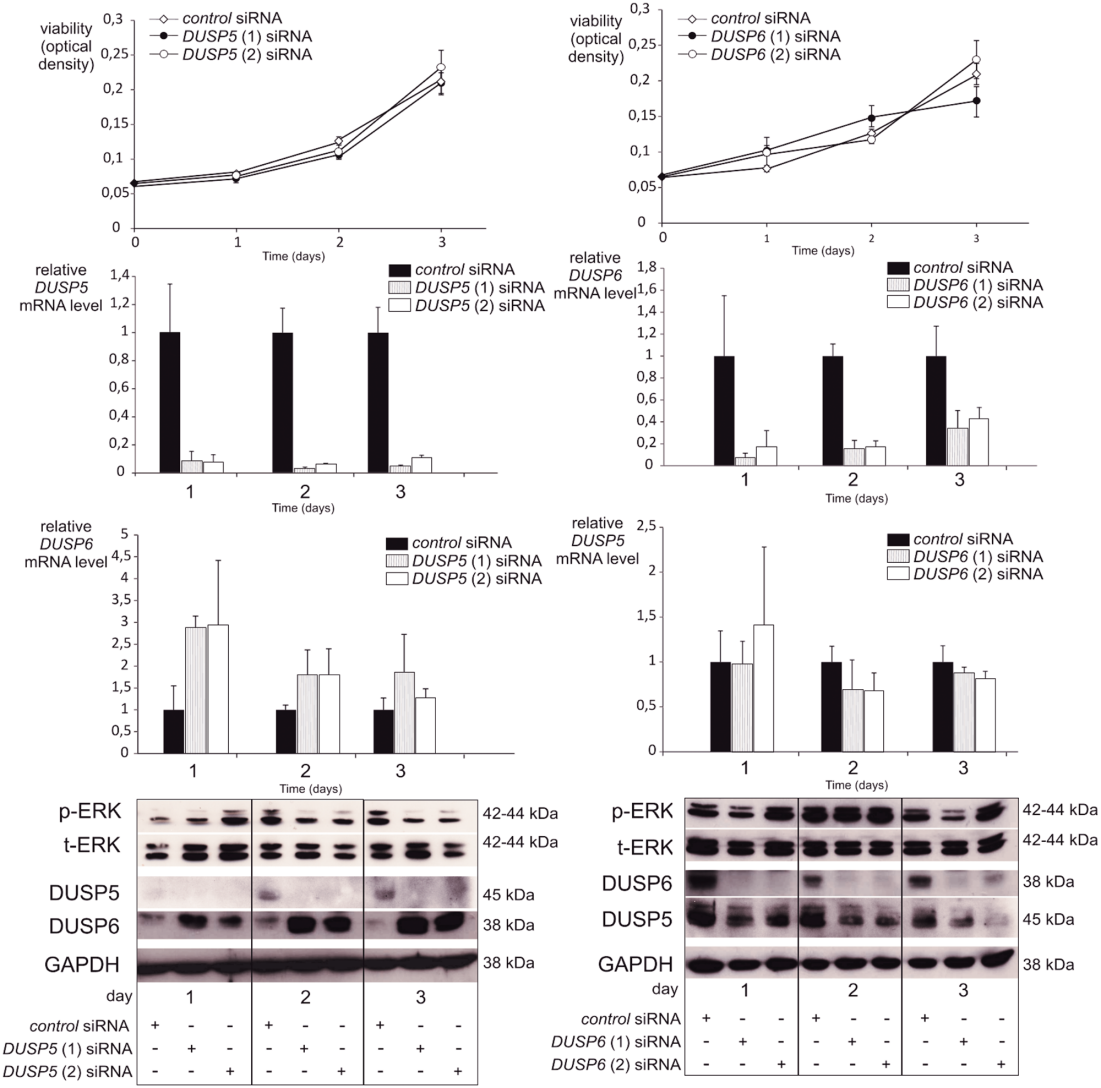
Silencing of DUSP5 or DUSP6 does not affect viability of 8505c BRAF^V600E^ cells. The 8505c cell line was transfected with the indicated siRNA. Cell viability was determined using the MTT assay the day of transection and for the following 3 days. Results are representative of at least three independent experiments. Gene knockdown was confirmed by reverse-transcription qPCR and Western blotting. DUSP5 protein level in control 8505c cells at day one was considered present, albeit weakly. Cells transfected with control siRNA at each time point were used as control. 8505c cells were also subjected to Western blotting using p-ERK, t-ERK and GAPDH antibodies.

**Fig 7:**
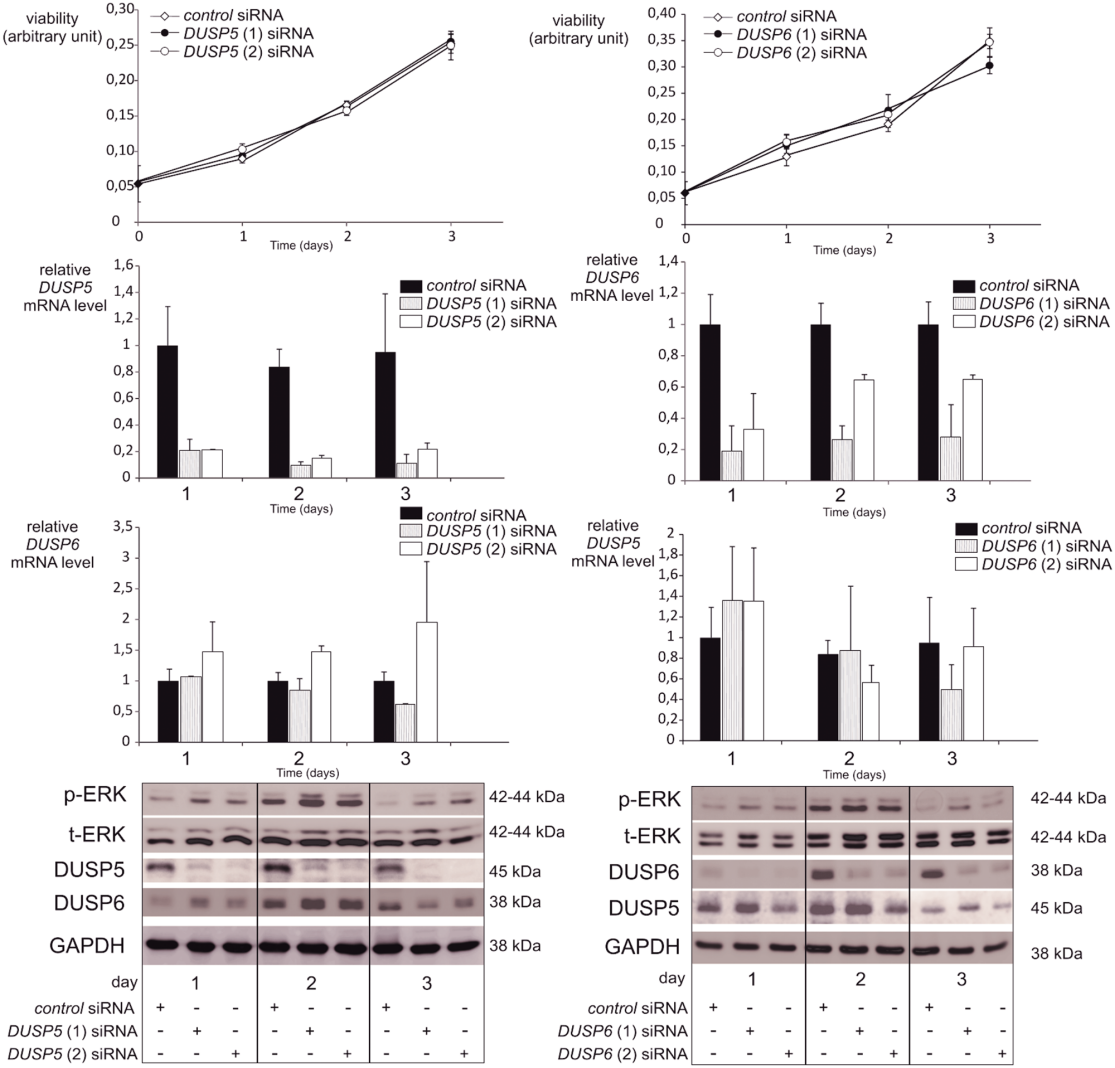
Silencing of DUSP5 or DUSP6 does not affect viability of BCPAP BRAF^V600E^ cells. The BCPAP cell line was transfected with the indicated siRNA. Cell viability was determined using the MTT assay the day of transection and for the following 3 days. Results are representative of at least three independent experiments. Gene knockdown was confirmed by reverse-transcription qPCR and Western blotting. Cells transfected with control siRNA at each time point were used as control. BCPAP cells were also subjected to Western blotting using p-ERK, t-ERK and GAPDH antibodies.

DUSP6 and DUSP5 act as natural terminators of MAPK signal transduction. Therefore, a tumor suppressive role could be hypothesized. Nevertheless, there are conflicting data in the literature regarding the role of such phosphatases on tumor cell proliferation [8, 31–33]. We investigated the proliferative effect of DUSP5 and DUSP6 silencing in two BRAF^V600E^ mutated human thyroid carcinoma cell lines (BCPAP and 8505c). Each siRNA effectively suppressed the targeted gene at the mRNA and protein level, but did not result in significant inhibition of cell viability (Figs 6 and 7), even five days after transfection (data not shown). Interestingly, inhibition of DUSP5 in the 8505c cell line was associated with a compensatory increase in DUSP6 mRNA and protein levels (Fig 6, left panel). This may explain the virtual lack of rise in the p-ERK levels while DUSP5 is inhibited (Fig 6). Conversely, the inhibition of DUSP6 in the 8505c cell line was associated with a decrease in DUSP5 protein and a slight decrease in mRNA levels after two days. Once again, p-ERK levels again were not modified.

**Fig 8:**
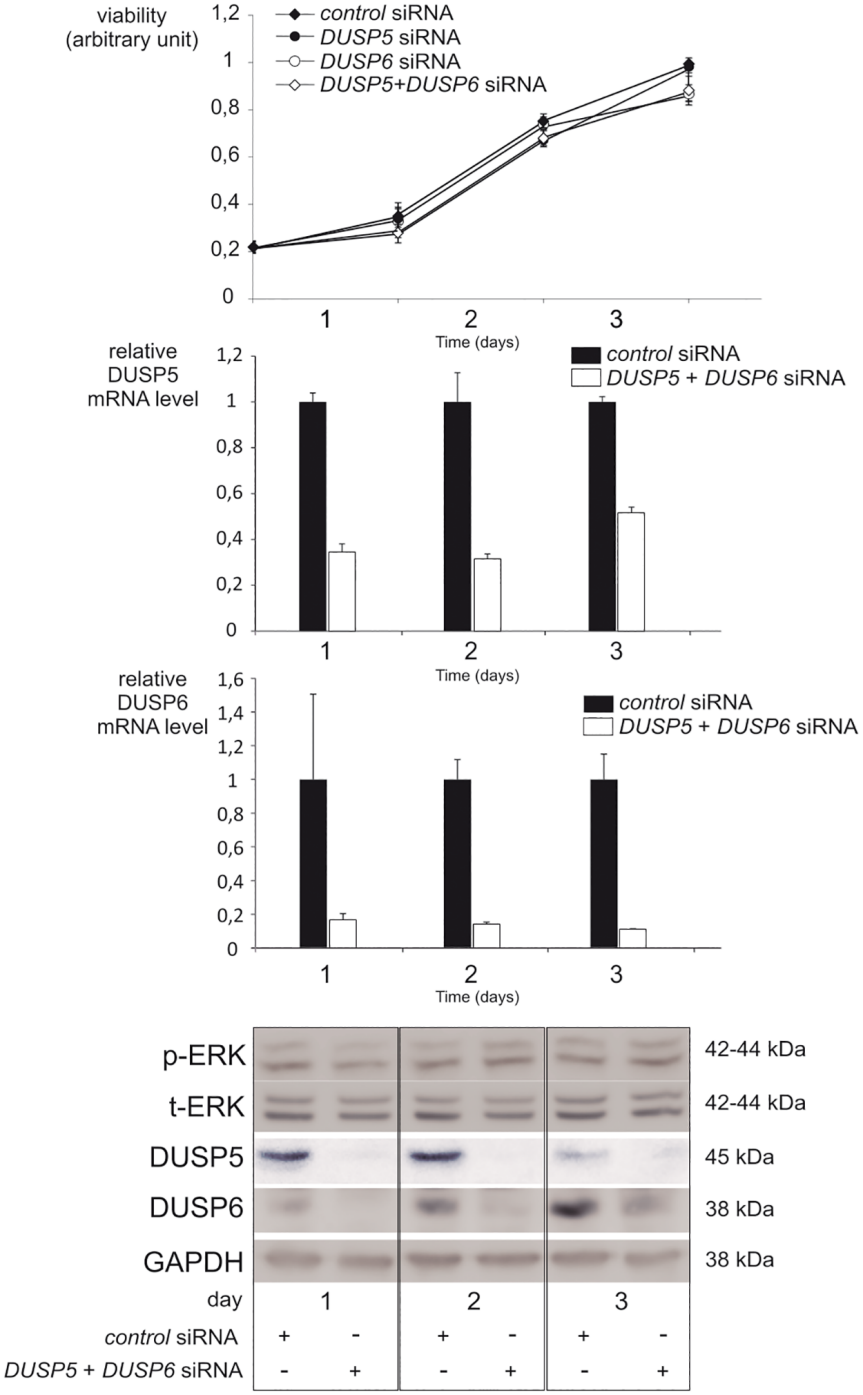
Simultaneous silencing of DUSP5 and DUSP6 does not affect viability of 8505c BRAF^V600E^ cells. Cells were transfected with the indicated siRNA. Cell viability was determined using the MTT assay the day of transfection and for the following 3 days. Results are representative of at least three independent experiments. Gene knockdown was confirmed by reverse-transcription qPCR and Western blotting. Cells transfected with control siRNA at each time point were used as control.

In the BCPAP cell line, inhibition of one DUSP was accompanied with an increasing tendency of the other at the mRNA level (Fig 7). To go further in testing a functional redundancy among this large phosphatase family, we decided to silence both DUSPs simultaneously in the 8505c cell line, which was confirmed at the mRNA and protein levels (Fig 8). The silencing of these two phosphatases did not either result in significant decrease in cell viability or increase in p-ERK levels.

### Silencing of DUSP5 or DUSP6 phosphatases by siRNA reduced the neoplastic properties of BRAF^V600E^-thyroid carcinoma cell lines.

**Fig 9:**
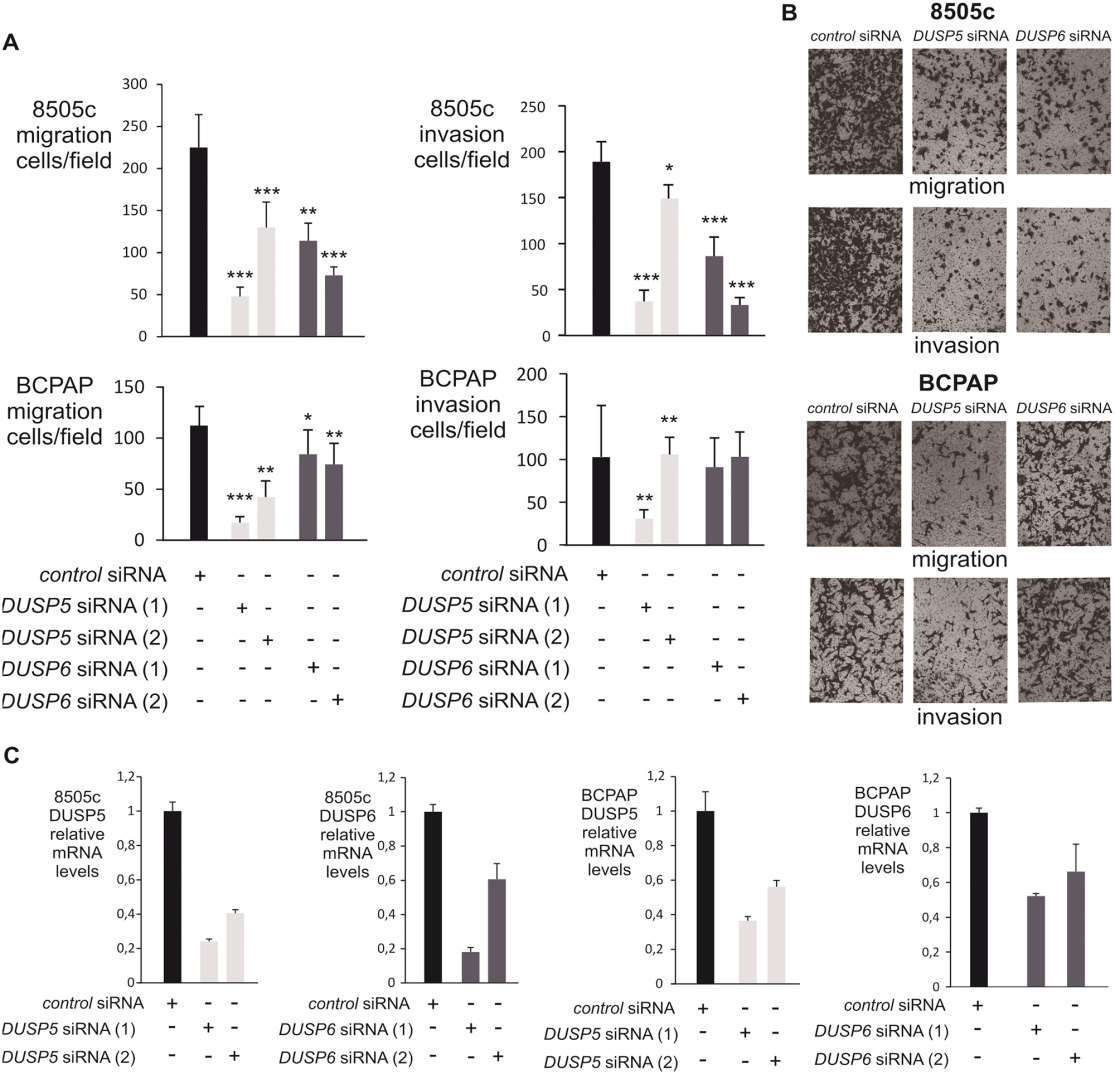
Effects of DUSP5 or DUSP6 silencing on migration and invasion of BCPAP and 8505c cells. (a). Forty-eight hours after transfection with the indicated siRNA migration and invasion were studied in the 8505c and BCPAP cell line. Assays were performed in triplicate, cells were counted in adjacent fields (n = 9). Data were reported as average cell number per field, and statistically compared with the control situation (unpaired t-test): *** *P* < 1. 10^4^; ** *P* < 1.10^−3^; * P < 0.05. (b) The most representative picture of migration and invasion for each siRNA is shown. (c). Gene knockdown was confirmed by reverse-transcription qPCR.

**Fig 10:**
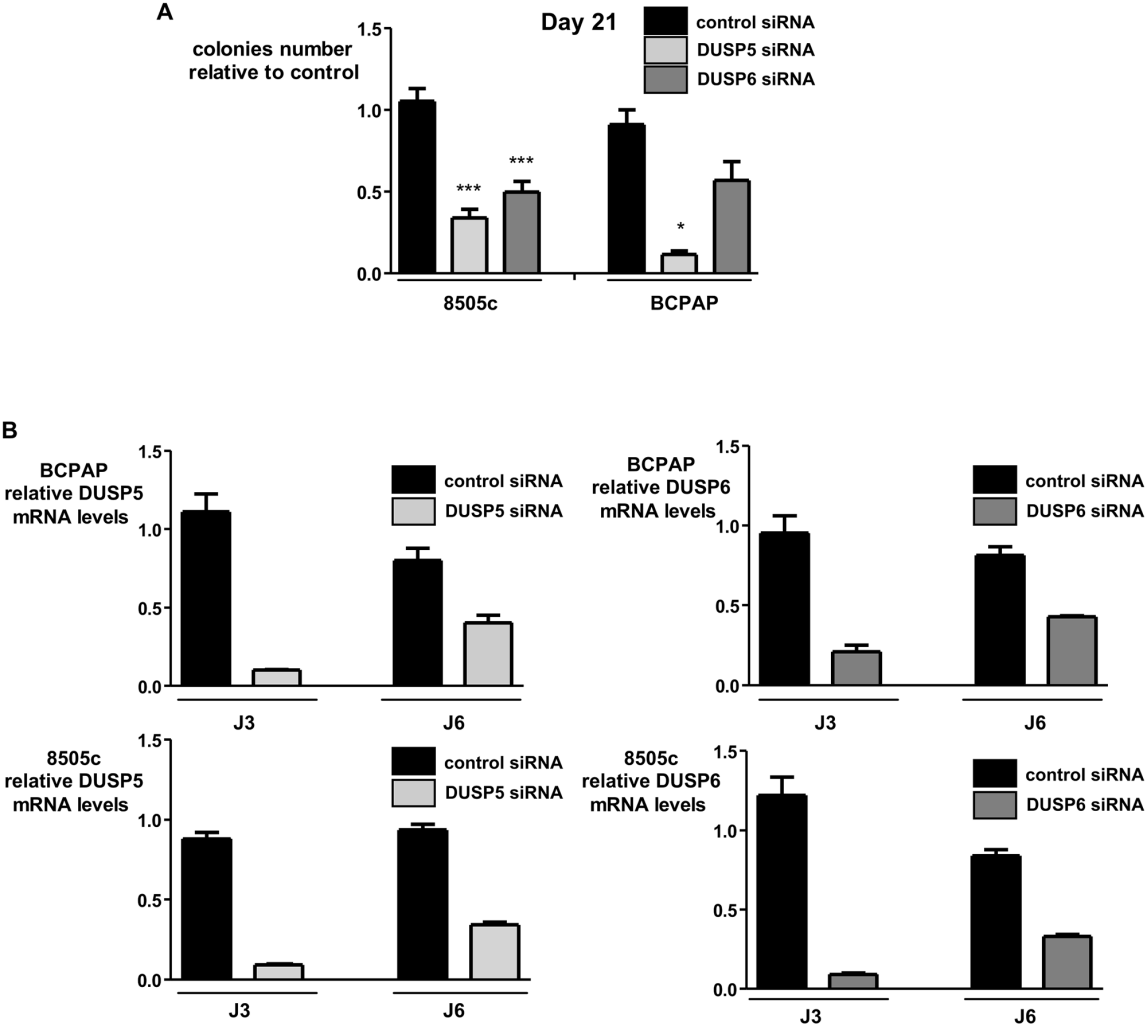
Effects of DUSP5 or DUSP6 silencing on the anchorage-independent growth of BCPAP and 8505c cells. (a). Twenty four hours after transfection with the indicated siRNA the anchorage-independent growth of the 8505c and BCPAP cell lines were analyzed in a soft agar assay. Colonies were counted microscopically at day 21. Values obtained by quantification of the colonies numbers are the mean of two independent experiments, and were then statistically compared with the control situation (student test): *** *P* < 1. 10^4^; * *P* < 0.05. (b) Gene knockdown was confirmed by reverse-transcription qPCR at 3 and 6 days after siRNA transfection.

Forty-eight hours after transfection with DUSP5 or DUSP6 siRNA the ability of 8505c to migrate into the lower chamber of a transwell or to invade the Matrigel Layer were significantly decreased (Fig 9a and 9b, upper panel). A significant decrease in the migration and invasion ability of BCPAP cells after DUSP5 silencing (Fig 9a and 9b, lower panel) was also observed. To resolve the differences observed between the two DUSP5 siRNA, we used a third DUSP5 siRNA and also observed a 50 to 90% reduction in the migration and invasion ability of both cell lines (S5 Fig). There is a trend, albeit less significant, to a decrease in the migration and invasion capacity of BCPAP cells after DUSP6 silencing (Fig 9a and 9b, lower panel). In each case DUSP5 and DUSP6 silencing was confirmed at the mRNA level (Fig 9c). In BCPAP and 8505c cells loss of DUSP5 and DUSP6 impaired the anchorage-independent cell growth in soft agar 21 days after DUSP5 or DUSP6 inactivation albeit with no statistical significance for DUSP6 siRNA in BCPAP cells (Fig 10a). A 80 to 90% reduction in the mRNA level of DUSP5 and DUSP6 was observed 3 days after transfection which remains at 60–70% at day 6 (Fig 10b).

## Discussion

Constitutive activation of the RAS-RAF-MEK-ERK pathway is thought to be mandatory to allow the growth of a PTC. BRAF^V600E^ mutation is associated with aggressive histo-pronostic features leading to disease recurrence, and even patient mortality in case of resistance to treatment. The molecular mechanisms underlying this aggressiveness are incompletely understood despite various hypotheses have been reported [2, 14].

In this paper, higher levels of p-MEK in human BRAF^V600E^ thyroid carcinoma cell lines in comparison with the others is in line with the conventional knowledge that the MAPK pathway is activated more robustly by the BRAF^V600E^ oncogene [29]. These *in vitro* differences in p-MEK levels in the cell lines were more difficult to observe with human thyroid tissue samples. Our analysis of TCGA proteomics data revealed that in BRAF^V600E^ PTCs p-MEK/t-MEK ratios were slightly higher (1.1 fold) than in BRAF wild-type PTCs, with the use of a highly sensitive technique, i.e. Reverse Phase Protein Lysate Microarray. Previous immunohistochemistry (IHC) data in human PTCs did not show significant differences in the levels of p-MEK between BRAF mutated and BRAF wild type PTCs, maybe reflecting the lack of sensitivity of IHC-based detection techniques for very small differences [4, 34].

The increasing p-MEK levels in BRAF^V600E^ PCCL3 cells during the kinetic confirmed the concept that the pathway is insensitive to upstream feedback inhibition. The observation that MEK kinase inhibition does not further induce p-MEK in BRAF^V600E^ PCCL3 and human thyroid cancer cells further supports this concept. On the opposite, p-MEK levels remained low during the kinetic in RET/PTC3 cells reflecting multiple negative feedback mechanisms upstream of MEK and accordingly, p-MEK levels were further induced after treatment with the MEK kinase inhibitor as well as in BRAF wild-type human thyroid cancer cells (release of negative feedback loop from ERK). Altogether, these results suggest that *in vitro* MEK phosphorylation but not ERK phosphorylation is a better marker of MAPK pathway output, as already shown in melanoma [35]. Despite this apparent similarity between the two tumor models, lineage-specific differences in effect of MAPK inhibition warrant specific studies for each model [36].

Increased MAPK pathway activation leads to an increased feedback inhibition at multiple levels of the kinase cascade to prevent overactivation of signaling output. As p-ERK levels were relatively constant from one human or rat thyroid cell lines to another, we sought to study DUSP5 and DUSP6 which act respectively in the nucleus and the cytoplasm to dephosphorylate ERK, to maintain its steady state levels.

We confirmed using two MEK inhibitors (UO126 and AZD6244) that *DUSP5* and *DUSP6* expression is regulated by the MEK-ERK pathway but not the PI3K pathway in PCCL3 cells and BRAF-mutated human PTC cell lines. *DUSP*5 and *DUSP6* overexpression in PTCs has been shown by gene expression profile studies [1, 12]. We confirmed here in a series of 39 human PTCs that *DUSP5* and *DUSP6* are overexpressed in PTCs compared to normal adjacent thyroid tissue. BRAF^V600E^ mutated PTCs expressed significantly higher levels of these phosphatases in comparison with other PTCs suggesting higher MAPK pathway activation, which is consistent with the recently published TCGA data [29]. Therefore *DUSP5* and *DUSP6* expression are markers of elevated output of the MAPK pathway.

Immunohistochemistry-based technique detection demonstrated that DUSP6 levels are increased in PTCs in comparison with benign thyroid neoplasms. Nevertheless, those levels were not correlated with BRAF^V600E^ mutation status [31]. The complex transcriptional, post-transcriptional and post-translational regulation of DUSP5 and DUSP6 may explain that reproducible correlation between MAPK pathway activation and DUSPs protein levels is hard to predict. In fact, accumulation of DUSP5 and DUSP6 protein upon ERK activation [6, 7, 37–40] is regulated by rapid proteasomal degradation [6, 7, 11, 40]. Moreover, ERK can phosphorylate DUSP6 on two different serines and subsequently enhances its proteasomal degradation [11]. As a consequence discrepancies between DUSP6 protein and mRNA levels have been frequently observed [6, 7, 39], which make the system even more complex to analyze.

It could be hypothesized that both phosphatases play a crucial role in tumorigenesis through the fine-tuning of magnitude and duration of ERK activation [41]. Absence of mutation of *DUSP5* and *DUSP6* reported in thyroid cancers [1] as well as in melanomas [42] support the hypothesis that these phosphatases have no tumor suppressor role in this type of carcinoma. We adopted RNA interference approach to evaluate the role of these phosphatases in PTCs. Consequences of the silencing of both phosphatases have never been studied in BRAF-mutated 8505c and BCPAP cell lines. We could not observe any effect on cell viability, even after prolonged silencing. We also report here for the first time the consequences of DUSP5 inactivation in human thyroid cancer cells. So far, the impact of DUSP5 inactivation in tumorigenesis has only been studied *in vitro* in human gastric and uterine cervix cancer cells and was responsible respectively for enhanced cell proliferation or massive apoptosis [32, 43]. *In vivo*, mice lacking DUSP5 show a greatly increased sensitivity to mutant Ras-driven papilloma formation in a pharmacological model of induced skin carcinogenesis [33]. In this model DUSP5 loss has no effect on (12-O-tetradecanoylphorbol-13-acetate) TPA-induced skin proliferation. Concerning DUSP6, we found no effect of its silencing on cell viability which is in apparent contradiction with previous report in BRAF^V600E^ and RET/PTC human thyroid cancer cell lines [13, 31]. However, the effect of these phosphatases on proliferation appears to be cell-type dependent [8]. Complex and opposite effects of DUSP in tumorigenesis have already been pointed out and reviewed recently by Caunt and Keyse [44]. While DUSP6 silencing with siRNA led to prompt (<72 hours) cell death through caspase activation in HeLa cells [32], on the opposite, it does not cause apoptosis in human thyroid TPC1 cells harboring *RET/PTC1* rearrangement. We report here for the first time the positive role of DUSP5 on *in vitro* cell migration and invasion in two BRAF^V600E^ thyroid tumor cell lines, as indicated by the decreased capacity of migration and invasion after DUSP5 invalidation in the 8505c and BCPAP cell lines. We found that DUSP6 silencing clearly reduced the invasion and migration ability of 8505c cells, in line with the results of Degl’Innocenti et al. who found reduced neoplastic properties of BRAF^V600E^ NIM-1 thyroid carcinoma cells after DUSP6 silencing [13]. While we found no significant effect of DUSP6 silencing on invasion capacity of BCPAP cells, contrary to Degl’innocenti et al. [13], we found reduced migration capacity of BCPAP cells after DUSP6 silencing, which was not studied by Degl’Innocenti et al. [13]. We also originally demonstrated that loss of DUSP5 and DUSP6 in BCPAP and 8505c cells impaired their anchorage-independent cell growth, in agreement with our results of the migration and invasion assays which are in favor of a pro-tumoral role of DUSP5 and DUSP6.

Pronounced compensatory increase in transcription of one DUSP when the other is knockdowned has been observed for *DUSP1* and *DUSP2* [45]. In our work DUSP5 inactivation is combined with a clear compensation by DUSP6 at the mRNA and protein levels which may explain the absence of consequence on proliferation and p-ERK levels. As previously demonstrated, a slight increase in the nuclear levels of p-ERK occurs upon inactivation of DUSP5, without any changes in p-ERK levels in the total extract [33]. This increase in the nuclear levels of p-ERK may explain the compensatory increase in DUSP6 mRNA and protein. The absence of consequences of a double inhibition of DUSP5 and DUSP6 on p-ERK levels and cell viability could suggest subtle changes in the levels of p-ERK occurring at the nuclear level only or a compensatory increase in other DUSPs or other phosphatases. Other members of the DUSP family that dephosphorylate ERK are induced by MAPK signaling (the nuclear DUSP1, DUSP2 and DUSP4; the cytosolic DUSP7 and DUSP9) [8] and are, thus, good candidate genes to study in order to understand the compensatory increase in this context. Subtle changes in the spatiotemporal regulation and activity of ERK induced by DUSP5 and DUSP6 inactivation may also explain the observed impact on invasion and migration in BCPAP and 8505 cells. However, we cannot formally exclude the possibility that DUSP5 and DUSP6 regulate tumor cell migration and invasion independently of ERK. In conclusion, our data confirmed that *DUSP5* and *DUSP6* mRNA levels are markers of activation of the MAPK signaling pathway in PTCs. Higher output of the MAPK pathway in BRAF^V600E^ mutated thyroid carcinomas provides an explanation for their poorer prognosis compared to other PTCs. DUSP5 and DUSP6 have no tumor suppressor properties in two BRAF^V600E^ thyroid carcinoma models, but instead they seem to have a protumorigenic role on thyroid carcinogenesis. The lack of effect in proliferation after specific inhibition of DUSP5 or DUSP6 or both DUSPs suggests redundancy and functional compensation in the large family of DUSP. Future work should focus on transcriptomic analysis of DUSPs expression modification, more specifically compensatory changes when one or several DUSPs are inactivated in human thyroid cancer cell lines.

## Supporting information

S1 Fig High phosphorylated MEK levels in serum-starved human thyroid carcinoma cell lines harboring the BRAF^V600E^ mutation. Eleven human thyroid cancer-derived cell lines were grown in 0.5% FBS medium for 48h and studied for MEK and ERK activation: two cell lines without known MAPK pathway genetic alteration, one with a *RET/PTC* rearrangement, two with a *RAS* activating point mutation, and five with the BRAF^V600E^ mutation. Protein expression levels were assayed by immunoblot for the BRAF^V600E^ mutation, phosphorylated MEK (p-MEK), total MEK1 (t-MEK1), phosphorylated ERK (p-ERK), total ERK (t-ERK) and GAPDH.
(TIF)

S2 Fig DUSP5 and DUSP6 levels in the eleven human thyroid carcinoma cell lines. Cells were maintained for 48h in 0.5% FBS medium. A *DUSP5* and *DUSP6* mRNA were then analyzed using real time Reverse-Transcription qPCR and normalized to cyclophilin mRNA levels. *DUSP5* and *DUSP6* mRNA levels in BCPAP cells were arbitrary set at 1. *DUSP6* mRNA levels in TTA1 cells were almost undetectable. B. Whole cell lysates of the eleven human thyroid carcinoma cell lines were subjected to immunoblotting with DUSP5 and DUSP6 antibodies.
(TIF)

S3 Fig No effect of doxycycline itself on phosphorylated ERK levels in non-transformed PCCL3 cells. (TIF)

S4 Fig BRAF^V600E^-mutated human thyroid carcinoma cells are insensitive to relief of ERK dependent upstream negative feedback. A. BRAF-mutated and BRAF-wild type human thyroid cancer cells were cultured in 0.5% FBS containing medium for 48h and then treated for 6 hours with the MEK inhibitor AZD6244. Whole cell extracts were subjected to the indicated antibodies. B. For each cell line the ratio pMEK/tMEK after AZD6244 treatment relative to the basal pMEK/tMEK ratio is represented.
(TIF)

S5 Fig Effects of a third *DUSP5* siRNA on migration and invasion in BCPAP and 8505c cells. (TIF)

## References

[1] Cancer Genome Atlas Research Network. Electronic address gue, Cancer Genome Atlas Research N. Integrated genomic characterization of papillary thyroid carcinoma. Cell. 2014;159(3):676–90. 25417114 10.1016/j.cell.2014.09.050 PMC4243044

[2] Xing M. BRAF mutation in papillary thyroid cancer: pathogenic role, molecular bases, and clinical implications. Endocr Rev. 2007;28(7):742–62. doi: 10.1210/er.2007-0007 . 17940185 10.1210/er.2007-0007

[3] Lito P, Pratilas CA, Joseph EW, Tadi M, Halilovic E, Zubrowski M, et al Relief of profound feedback inhibition of mitogenic signaling by RAF inhibitors attenuates their activity in BRAFV600E melanomas. Cancer cell. 2012;22(5):668–82. doi: 10.1016/j.ccr.2012.10.009 ;. 23153539 10.1016/j.ccr.2012.10.009 PMC3713778

[4] Mitsiades CS, Negri J, McMullan C, McMillin DW, Sozopoulos E, Fanourakis G, et al Targeting BRAFV600E in thyroid carcinoma: therapeutic implications. Molecular cancer therapeutics. 2007;6(3):1070–8. doi: 10.1158/1535-7163.MCT-06-0449 . 17363500 10.1158/1535-7163.MCT-06-0449

[5] Zuo H, Nakamura Y, Yasuoka H, Zhang P, Nakamura M, Mori I, et al Lack of association between BRAF V600E mutation and mitogen-activated protein kinase activation in papillary thyroid carcinoma. Pathology international. 2007;57(1):12–20. doi: 10.1111/j.1440-1827.2007.02050.x . 17199737 10.1111/j.1440-1827.2007.02050.x

[6] Kucharska A, Rushworth LK, Staples C, Morrice NA, Keyse SM. Regulation of the inducible nuclear dual-specificity phosphatase DUSP5 by ERK MAPK. Cell Signal. 2009;21(12):1794–805. doi: 10.1016/j.cellsig.2009.07.015 . 19666109 10.1016/j.cellsig.2009.07.015

[7] Zeliadt NA, Mauro LJ, Wattenberg EV. Reciprocal regulation of extracellular signal regulated kinase 1/2 and mitogen activated protein kinase phosphatase-3. Toxicol Appl Pharmacol. 2008;232(3):408–17. doi: 10.1016/j.taap.2008.08.007 . 18771677 10.1016/j.taap.2008.08.007 PMC2581931

[8] Nunes-Xavier C, Roma-Mateo C, Rios P, Tarrega C, Cejudo-Marin R, Tabernero L, et al Dual-specificity MAP kinase phosphatases as targets of cancer treatment. Anti-cancer agents in medicinal chemistry. 2011;11(1):109–32. . 21288197 10.2174/187152011794941190

[9] Mandl M, Slack DN, Keyse SM. Specific inactivation and nuclear anchoring of extracellular signal-regulated kinase 2 by the inducible dual-specificity protein phosphatase DUSP5. Mol Cell Biol. 2005;25(5):1830–45. doi: 10.1128/MCB.25.5.1830-1845.2005 . 15713638 10.1128/MCB.25.5.1830-1845.2005 PMC549372

[10] Karlsson M, Mathers J, Dickinson RJ, Mandl M, Keyse SM. Both nuclear-cytoplasmic shuttling of the dual specificity phosphatase MKP-3 and its ability to anchor MAP kinase in the cytoplasm are mediated by a conserved nuclear export signal. J Biol Chem. 2004;279(40):41882–91. doi: 10.1074/jbc.M406720200 . 15269220 10.1074/jbc.M406720200

[11] Marchetti S, Gimond C, Chambard JC, Touboul T, Roux D, Pouyssegur J, et al Extracellular signal-regulated kinases phosphorylate mitogen-activated protein kinase phosphatase 3/DUSP6 at serines 159 and 197, two sites critical for its proteasomal degradation. Mol Cell Biol. 2005;25(2):854–64. doi: 10.1128/MCB.25.2.854-864.2005 . 15632084 10.1128/MCB.25.2.854-864.2005 PMC543408

[12] Griffith OL, Melck A, Jones SJ, Wiseman SM. Meta-analysis and meta-review of thyroid cancer gene expression profiling studies identifies important diagnostic biomarkers. J Clin Oncol. 2006;24(31):5043–51. doi: 10.1200/JCO.2006.06.7330 . 17075124 10.1200/JCO.2006.06.7330

[13] Degl'Innocenti D, Romeo P, Tarantino E, Sensi M, Cassinelli G, Catalano V, et al DUSP6/MKP3 is overexpressed in papillary and poorly differentiated thyroid carcinoma and contributes to neoplastic properties of thyroid cancer cells. Endocrine-related cancer. 2013;20(1):23–37. doi: 10.1530/ERC-12-0078 . 23132790 10.1530/ERC-12-0078

[14] Mitsutake N, Knauf JA, Mitsutake S, Mesa C Jr., Zhang L, Fagin JA. Conditional BRAFV600E expression induces DNA synthesis, apoptosis, dedifferentiation, and chromosomal instability in thyroid PCCL3 cells. Cancer Res. 2005;65(6):2465–73. doi: 10.1158/0008-5472.CAN-04-3314 . 15781663 10.1158/0008-5472.CAN-04-3314

[15] Shirokawa JM, Elisei R, Knauf JA, Hara T, Wang J, Saavedra HI, et al Conditional apoptosis induced by oncogenic ras in thyroid cells. Mol Endocrinol. 2000;14(11):1725–38. doi: 10.1210/mend.14.11.0559 . 11075808 10.1210/mend.14.11.0559

[16] Wang J, Knauf JA, Basu S, Puxeddu E, Kuroda H, Santoro M, et al Conditional expression of RET/PTC induces a weak oncogenic drive in thyroid PCCL3 cells and inhibits thyrotropin action at multiple levels. Mol Endocrinol. 2003;17(7):1425–36. doi: 10.1210/me.2003-0041 . 12690093 10.1210/me.2003-0041

[17] Heldin NE, Cvejic D, Smeds S, Westermark B. Coexpression of functionally active receptors for thyrotropin and platelet-derived growth factor in human thyroid carcinoma cells. Endocrinology. 1991;129(4):2187–93. doi: 10.1210/endo-129-4-2187 . 1655394 10.1210/endo-129-4-2187

[18] Gustavsson B, Hermansson A, Andersson AC, Grimelius L, Bergh J, Westermark B, et al Decreased growth rate and tumour formation of human anaplastic thyroid carcinoma cells transfected with a human thyrotropin receptor cDNA in NMRI nude mice treated with propylthiouracil. Mol Cell Endocrinol. 1996;121(2):143–51. . 8892315 10.1016/0303-7207(96)03859-2

[19] Xu X, Quiros RM, Gattuso P, Ain KB, Prinz RA. High prevalence of BRAF gene mutation in papillary thyroid carcinomas and thyroid tumor cell lines. Cancer Res. 2003;63(15):4561–7. . 12907632

[20] Yano Y, Kamma H, Matsumoto H, Fujiwara M, Bando H, Hara H, et al Growth suppression of thyroid cancer cells by adenylcyclase activator. Oncol Rep. 2007;18(2):441–5. . 17611668

[21] Chung SH, Onoda N, Ishikawa T, Ogisawa K, Takenaka C, Yano Y, et al Peroxisome proliferator-activated receptor gamma activation induces cell cycle arrest via the p53-independent pathway in human anaplastic thyroid cancer cells. Jpn J Cancer Res. 2002;93(12):1358–65. . 12495476 10.1111/j.1349-7006.2002.tb01245.x PMC5926938

[22] Tanaka J, Ogura T, Sato H, Hatano M. Establishment and biological characterization of an in vitro human cytomegalovirus latency model. Virology. 1987;161(1):62–72. . 2823470 10.1016/0042-6822(87)90171-1

[23] Gioanni J, Zanghellini E, Mazeau C, Zhang D, Courdi A, Farges M, et al [Characterization of a human cell line from an anaplastic carcinoma of the thyroid gland]. Bull Cancer. 1991;78(11):1053–62. . 1369551

[24] Salerno P, De Falco V, Tamburrino A, Nappi TC, Vecchio G, Schweppe RE, et al Cytostatic activity of adenosine triphosphate-competitive kinase inhibitors in BRAF mutant thyroid carcinoma cells. J Clin Endocrinol Metab. 2010;95(1):450–5. doi: 10.1210/jc.2009-0373 . 19880792 10.1210/jc.2009-0373

[25] Kurebayashi J, Tanaka K, Otsuki T, Moriya T, Kunisue H, Uno M, et al All-trans-retinoic acid modulates expression levels of thyroglobulin and cytokines in a new human poorly differentiated papillary thyroid carcinoma cell line, KTC-1. J Clin Endocrinol Metab. 2000;85(8):2889–96. doi: 10.1210/jcem.85.8.6732 . 10946899 10.1210/jcem.85.8.6732

[26] Lee JJ, Foukakis T, Hashemi J, Grimelius L, Heldin NE, Wallin G, et al Molecular cytogenetic profiles of novel and established human anaplastic thyroid carcinoma models. Thyroid. 2007;17(4):289–301. doi: 10.1089/thy.2006.0246 . 17465858 10.1089/thy.2006.0246

[27] Schweppe RE, Klopper JP, Korch C, Pugazhenthi U, Benezra M, Knauf JA, et al Deoxyribonucleic acid profiling analysis of 40 human thyroid cancer cell lines reveals cross-contamination resulting in cell line redundancy and misidentification. J Clin Endocrinol Metab. 2008;93(11):4331–41. doi: 10.1210/jc.2008-1102 . 18713817 10.1210/jc.2008-1102 PMC2582569

[28] Ricarte-Filho JC, Li S, Garcia-Rendueles ME, Montero-Conde C, Voza F, Knauf JA, et al Identification of kinase fusion oncogenes in post-Chernobyl radiation-induced thyroid cancers. The Journal of clinical investigation. 2013;123(11):4935–44. doi: 10.1172/JCI69766 ;. 24135138 10.1172/JCI69766 PMC3809792

[29] Cancer Genome Atlas Research N. Integrated genomic characterization of papillary thyroid carcinoma. Cell. 2014;159(3):676–90. doi: 10.1016/j.cell.2014.09.050 ;. 25417114 10.1016/j.cell.2014.09.050 PMC4243044

[30] Yeh TC, Marsh V, Bernat BA, Ballard J, Colwell H, Evans RJ, et al Biological characterization of ARRY-142886 (AZD6244), a potent, highly selective mitogen-activated protein kinase kinase 1/2 inhibitor. Clinical cancer research: an official journal of the American Association for Cancer Research. 2007;13(5):1576–83. doi: 10.1158/1078-0432.CCR-06-1150 . 17332304 10.1158/1078-0432.CCR-06-1150

[31] Lee JU, Huang S, Lee MH, Lee SE, Ryu MJ, Kim SJ, et al Dual specificity phosphatase 6 as a predictor of invasiveness in papillary thyroid cancer. European journal of endocrinology / European Federation of Endocrine Societies. 2012;167(1):93–101. doi: 10.1530/EJE-12-0010 . 22535643 10.1530/EJE-12-0010

[32] MacKeigan JP, Murphy LO, Blenis J. Sensitized RNAi screen of human kinases and phosphatases identifies new regulators of apoptosis and chemoresistance. Nat Cell Biol. 2005;7(6):591–600. doi: 10.1038/ncb1258 . 15864305 10.1038/ncb1258

[33] Rushworth LK, Kidger AM, Delavaine L, Stewart G, van Schelven S, Davidson J, et al Dual-specificity phosphatase 5 regulates nuclear ERK activity and suppresses skin cancer by inhibiting mutant Harvey-Ras (HRasQ61L)-driven SerpinB2 expression. Proc Natl Acad Sci U S A. 2014;111(51):18267–72. doi: 10.1073/pnas.1420159112 ;. 25489104 10.1073/pnas.1420159112 PMC4280588

[34] Dultz LA, Dhar S, Ogilvie JB, Heller KS, Bar-Sagi D, Patel KN. Clinical and therapeutic implications of Sprouty2 feedback dysregulation in BRAF V600E-mutation-positive papillary thyroid cancer. Surgery. 2013;154(6):1239–44; discussion 44–5. doi: 10.1016/j.surg.2013.06.024 . 24094449 10.1016/j.surg.2013.06.024 PMC4100696

[35] Pratilas CA, Taylor BS, Ye Q, Viale A, Sander C, Solit DB, et al (V600E)BRAF is associated with disabled feedback inhibition of RAF-MEK signaling and elevated transcriptional output of the pathway. Proc Natl Acad Sci U S A. 2009;106(11):4519–24. doi: 10.1073/pnas.0900780106 . 19251651 10.1073/pnas.0900780106 PMC2649208

[36] Montero-Conde C, Ruiz-Llorente S, Dominguez JM, Knauf JA, Viale A, Sherman EJ, et al Relief of feedback inhibition of HER3 transcription by RAF and MEK inhibitors attenuates their antitumor effects in BRAF-mutant thyroid carcinomas. Cancer discovery. 2013;3(5):520–33. doi: 10.1158/2159-8290.CD-12-0531 ;. 23365119 10.1158/2159-8290.CD-12-0531 PMC3651738

[37] Bellou S, Hink MA, Bagli E, Panopoulou E, Bastiaens PI, Murphy C, et al VEGF autoregulates its proliferative and migratory ERK1/2 and p38 cascades by enhancing the expression of DUSP1 and DUSP5 phosphatases in endothelial cells. Am J Physiol Cell Physiol. 2009;297(6):C1477–89. doi: 10.1152/ajpcell.00058.2009 . 19741200 10.1152/ajpcell.00058.2009

[38] Buffet C, Catelli MG, Hecale-Perlemoine K, Bricaire L, Garcia C, Gallet-Dierick A, et al Dual Specificity Phosphatase 5, a Specific Negative Regulator of ERK Signaling, Is Induced by Serum Response Factor and Elk-1 Transcription Factor. PloS one. 2015;10(12):e0145484 doi: 10.1371/journal.pone.0145484 . 26691724 10.1371/journal.pone.0145484 PMC4687125

[39] Ekerot M, Stavridis MP, Delavaine L, Mitchell MP, Staples C, Owens DM, et al Negative-feedback regulation of FGF signalling by DUSP6/MKP-3 is driven by ERK1/2 and mediated by Ets factor binding to a conserved site within the DUSP6/MKP-3 gene promoter. Biochem J. 2008;412(2):287–98. doi: 10.1042/BJ20071512 . 18321244 10.1042/BJ20071512 PMC2474557

[40] Zhang Z, Kobayashi S, Borczuk AC, Leidner RS, Laframboise T, Levine AD, et al Dual specificity phosphatase 6 (DUSP6) is an ETS-regulated negative feedback mediator of oncogenic ERK signaling in lung cancer cells. Carcinogenesis. 2010;31(4):577–86. doi: 10.1093/carcin/bgq020 . 20097731 10.1093/carcin/bgq020 PMC2847094

[41] Bluthgen N, Legewie S, Kielbasa SM, Schramme A, Tchernitsa O, Keil J, et al A systems biological approach suggests that transcriptional feedback regulation by dual-specificity phosphatase 6 shapes extracellular signal-related kinase activity in RAS-transformed fibroblasts. The FEBS journal. 2009;276(4):1024–35. doi: 10.1111/j.1742-4658.2008.06846.x . 19154344 10.1111/j.1742-4658.2008.06846.x

[42] Hodis E, Watson IR, Kryukov GV, Arold ST, Imielinski M, Theurillat JP, et al A landscape of driver mutations in melanoma. Cell. 2012;150(2):251–63. doi: 10.1016/j.cell.2012.06.024 ;. 22817889 10.1016/j.cell.2012.06.024 PMC3600117

[43] Shin SH, Park SY, Kang GH. Down-regulation of dual-specificity phosphatase 5 in gastric cancer by promoter CpG island hypermethylation and its potential role in carcinogenesis. Am J Pathol. 2013;182(4):1275–85. doi: 10.1016/j.ajpath.2013.01.004 . 23402999 10.1016/j.ajpath.2013.01.004

[44] Caunt CJ, Keyse SM. Dual-specificity MAP kinase phosphatases (MKPs): shaping the outcome of MAP kinase signalling. The FEBS journal. 2013;280(2):489–504. doi: 10.1111/j.1742-4658.2012.08716.x ;. 22812510 10.1111/j.1742-4658.2012.08716.x PMC3594966

[45] Caunt CJ, Armstrong SP, Rivers CA, Norman MR, McArdle CA. Spatiotemporal regulation of ERK2 by dual specificity phosphatases. J Biol Chem. 2008;283(39):26612–23. doi: 10.1074/jbc.M801500200 ;. 18650424 10.1074/jbc.M801500200 PMC2546534

