# Supplementary figures and images for: DUSP5 and DUSP6, two ERK specific phosphatases, are markers of a higher MAPK signaling activation in BRAF mutated thyroid cancers
Source: PLoS One. 2017 Sep 14;12(9):e0184861. doi: 10.1371/journal.pone.0184861 (PMC5599027; doi:10.1371/journal.pone.0184861)

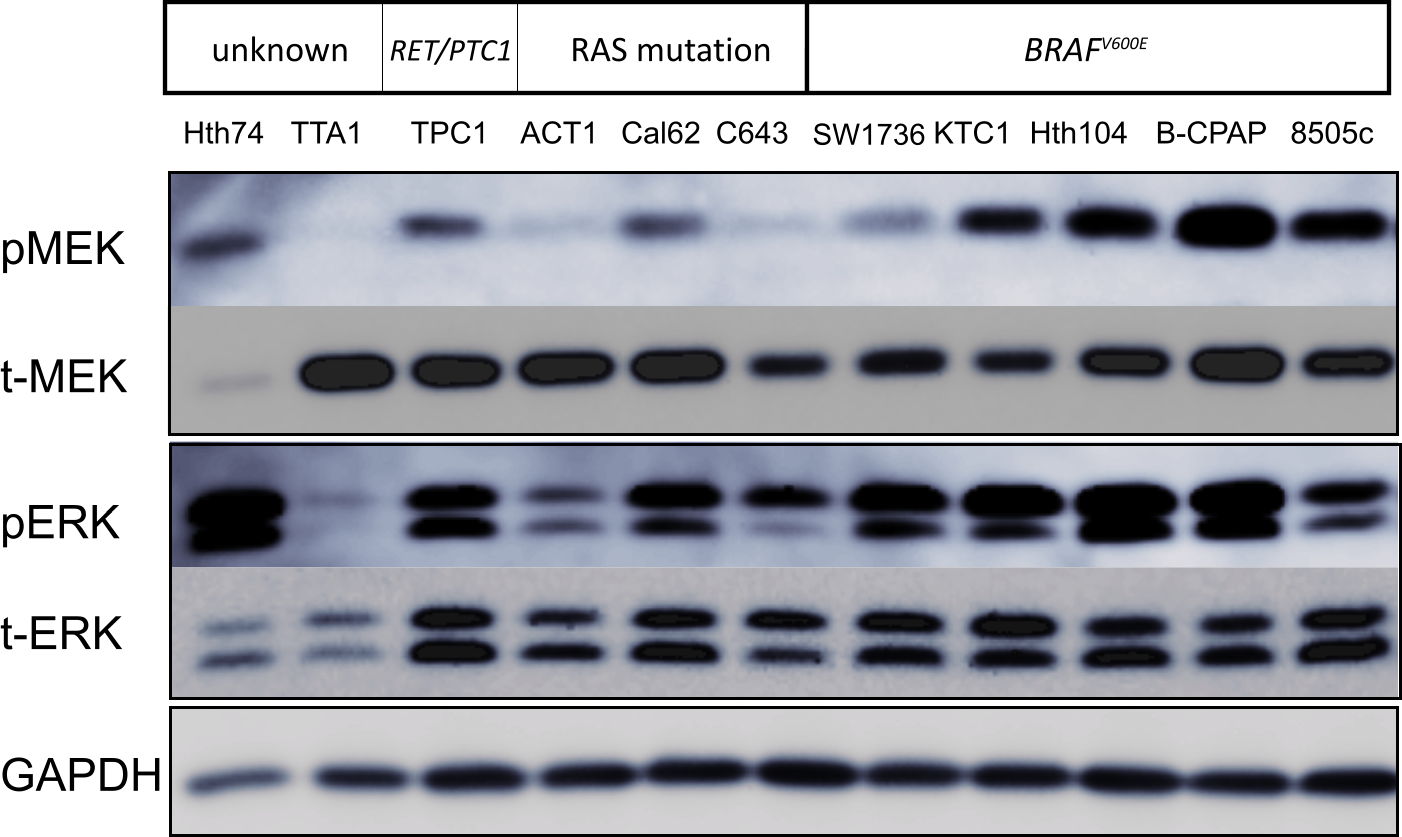

Supplement: S1 Fig — Eleven human thyroid cancer-derived cell lines were grown in 0.5% FBS medium for 48h and studied for MEK and ERK activation: two cell lines without known MAPK pathway genetic alteration, one with a RET/PTC rearrangement, two with a RAS activating point mutation, and five with the BRAFV600E mutation. Protein expression levels were assayed by immunoblot for the BRAFV600E mutation, phosphorylated MEK (p-MEK), total MEK1 (t-MEK1), phosphorylated ERK (p-ERK), total ERK (t-ERK) and GAPDH. (TIF) [file pone.0184861.s001.tif]

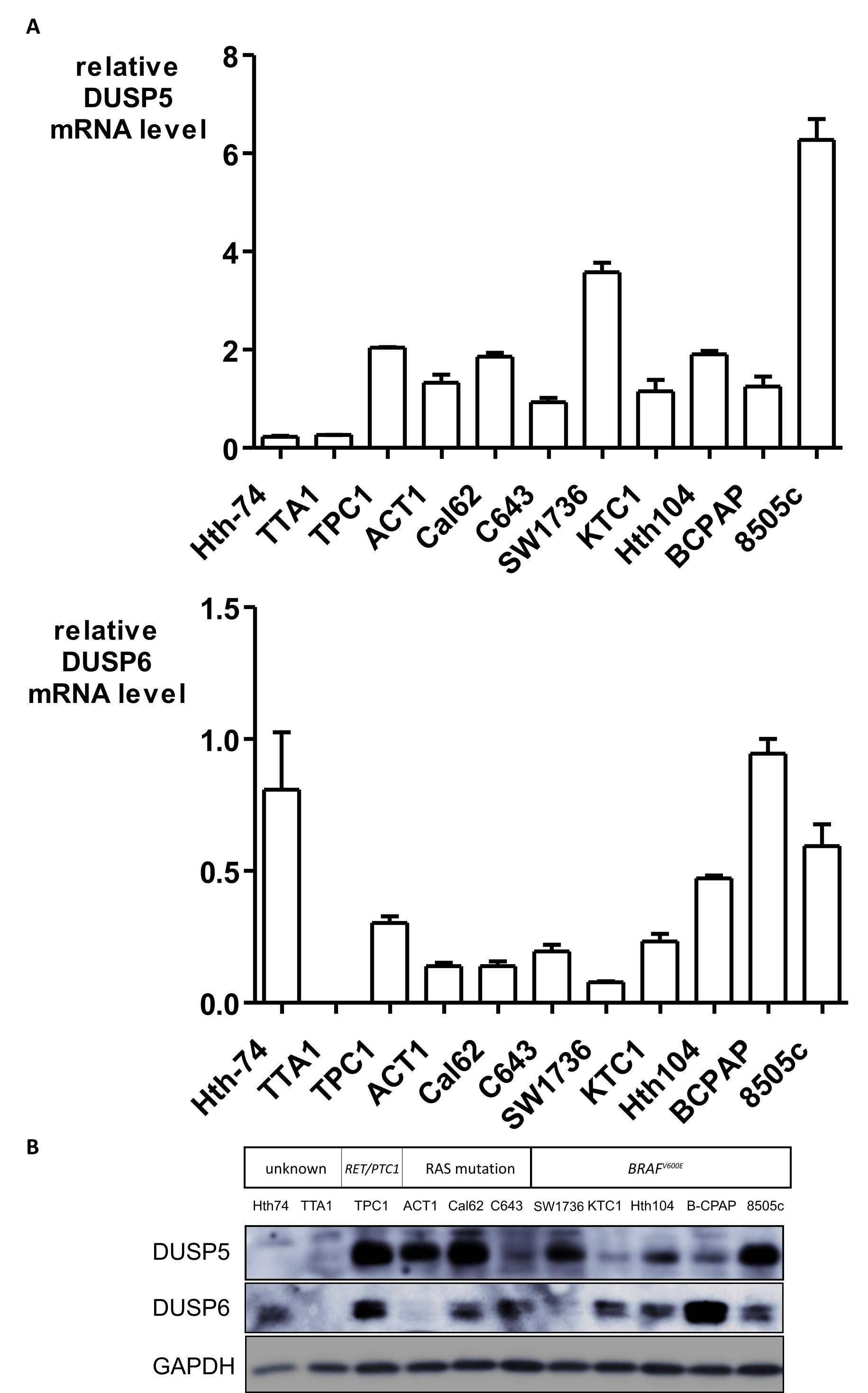

Supplement: S2 Fig — Cells were maintained for 48h in 0.5% FBS medium. A DUSP5 and DUSP6 mRNA were then analyzed using real time Reverse-Transcription qPCR and normalized to cyclophilin mRNA levels. DUSP5 and DUSP6 mRNA levels in BCPAP cells were arbitrary set at 1. DUSP6 mRNA levels in TTA1 cells were almost undetectable. B. Whole cell lysates of the eleven human thyroid carcinoma cell lines were subjected to immunoblotting with DUSP5 and DUSP6 antibodies. (TIF) [file pone.0184861.s002.tif]

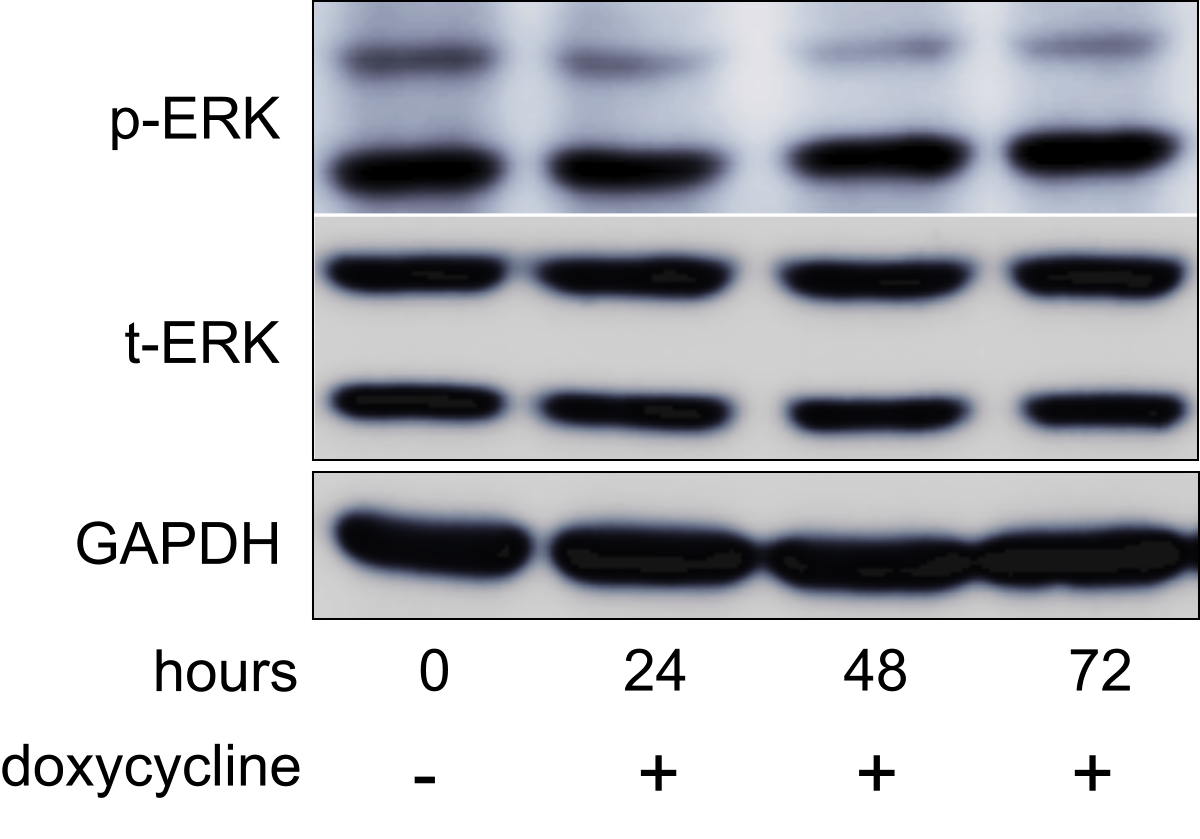

Supplement: S3 Fig — (TIF) [file pone.0184861.s003.tif]

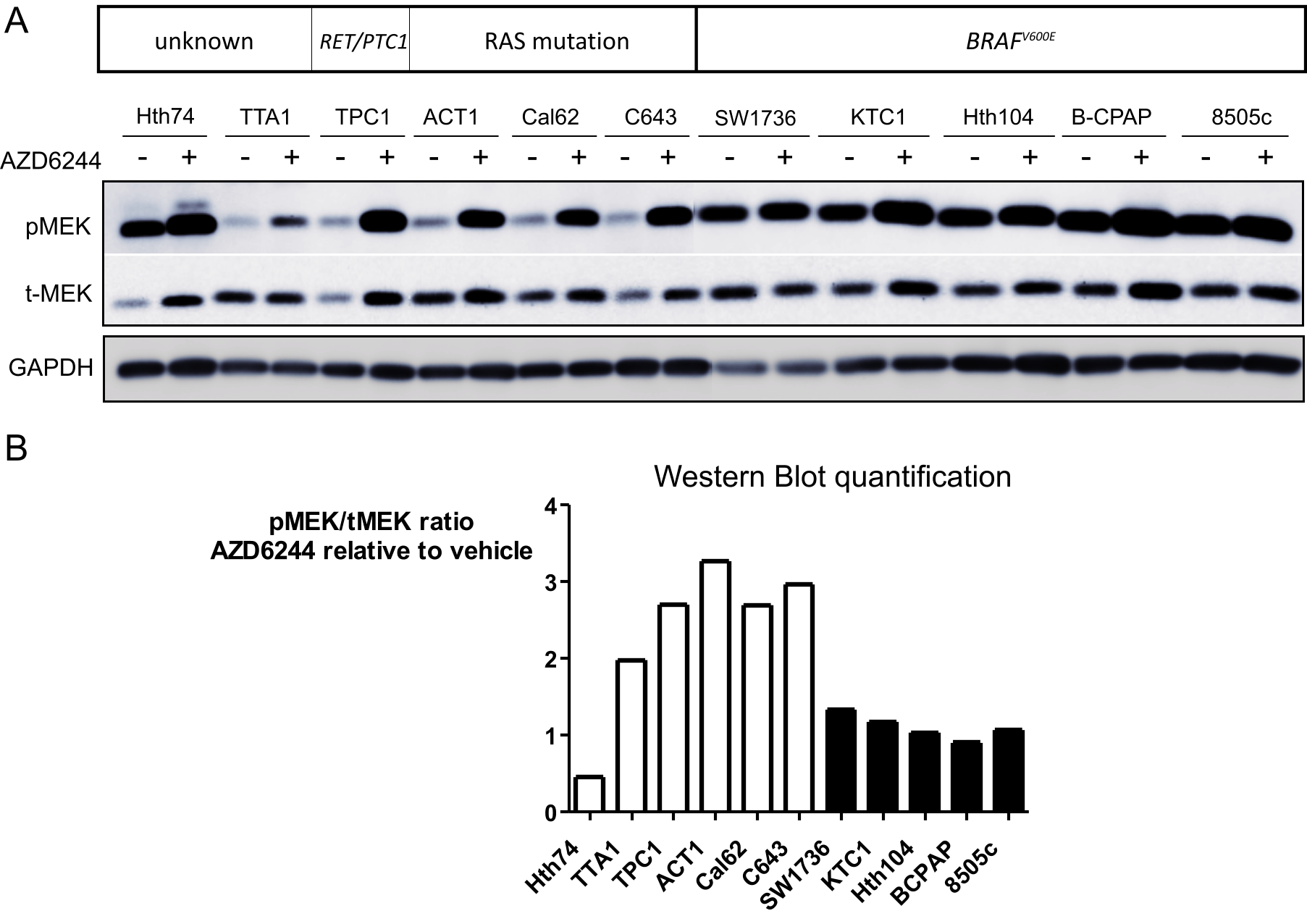

Supplement: S4 Fig — A. BRAF-mutated and BRAF-wild type human thyroid cancer cells were cultured in 0.5% FBS containing medium for 48h and then treated for 6 hours with the MEK inhibitor AZD6244. Whole cell extracts were subjected to the indicated antibodies. B. For each cell line the ratio pMEK/tMEK after AZD6244 treatment relative to the basal pMEK/tMEK ratio is represented. (TIF) [file pone.0184861.s004.tif]

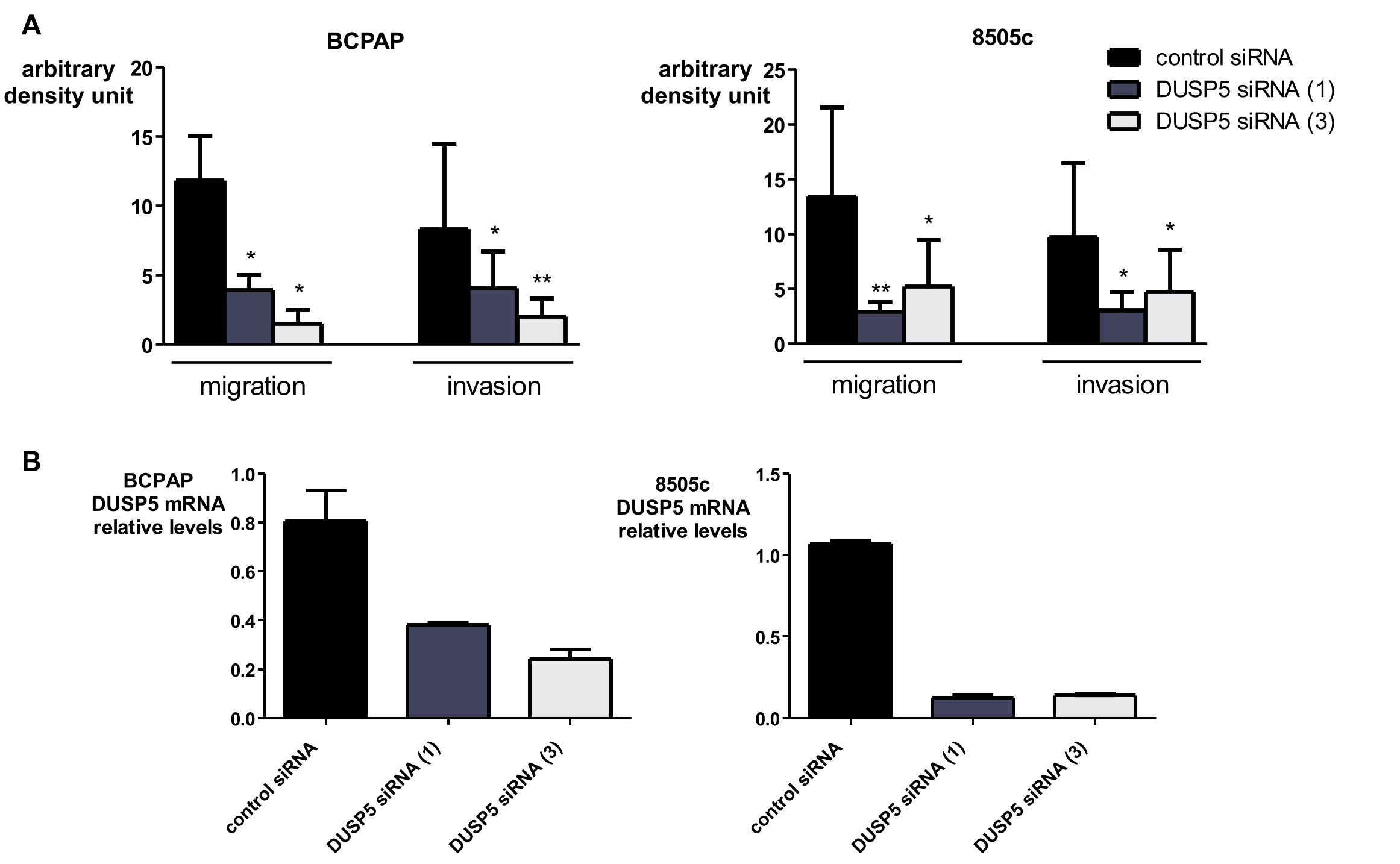

Supplement: S5 Fig — (TIF) [file pone.0184861.s005.tif]
